# Supplementary material for: Biomimetic superabsorbent hydrogel acts as a gut protective dynamic exoskeleton improving metabolic parameters and expanding A. muciniphila
Source: Cell Rep Med. 2023 Oct 17;4(10):101235. doi: 10.1016/j.xcrm.2023.101235 (PMC10591066; doi:10.1016/j.xcrm.2023.101235)
Supplement: Document S1. Figures S1–S6, Tables S1 and S2 [file mmc1.pdf]

Cell Reports Medicine, Volume *4*

## Supplemental information

# Biomimetic superabsorbent hydrogel acts as a gut protective dynamic exoskeleton improving metabolic parameters and expanding *A. muciniphila*

**Alessandra Silvestri, Antonio Gil-Gomez, Milena Vitale, Daniele Braga, Christian Demitri, Paola Brescia, Marta Madaghiele, Ilaria Spadoni, Bryan Jones, Giulia Fornasa, Juliette Mouries, Sara Carloni, Michela Lizier, Manuel Romero-Gomez, Giuseppe Penna, Alessandro Sannino, and Maria Rescigno**

# SUPPLEMENTARY MATERIAL

## Figure S1

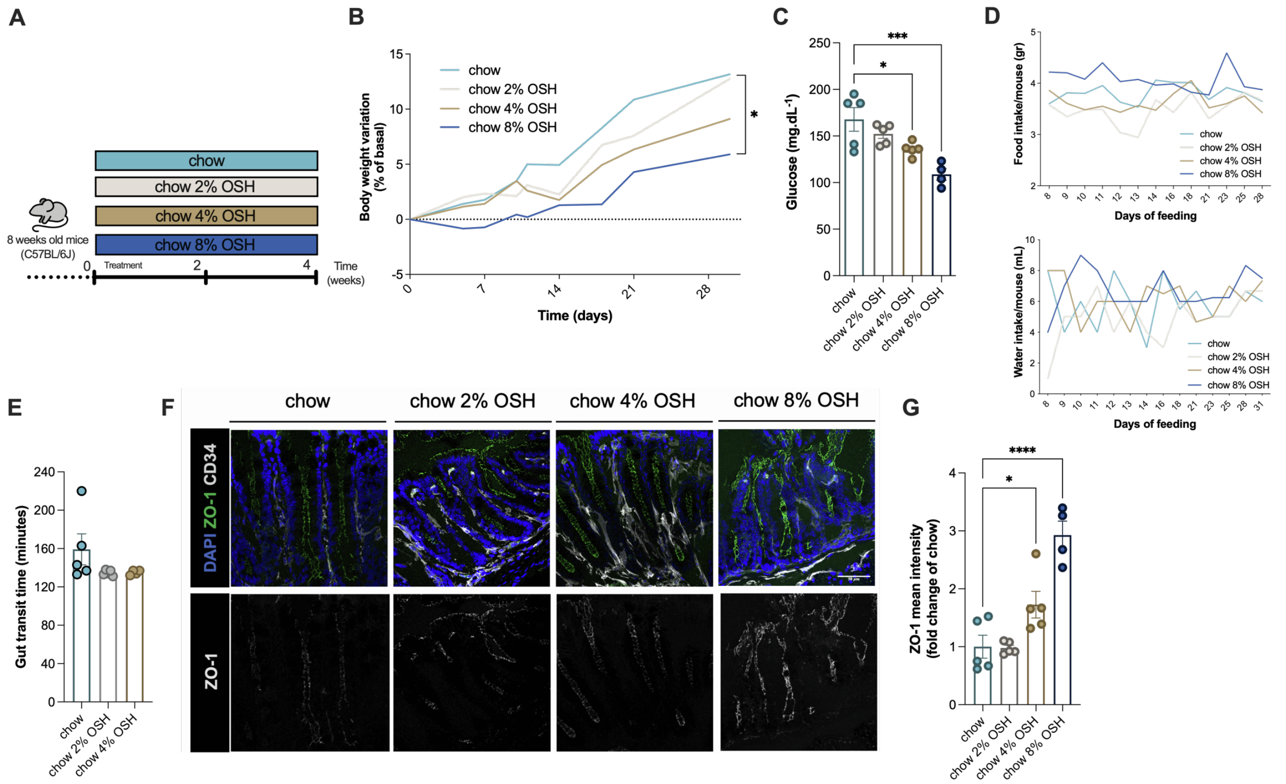


***Supplementary Figure 1*****. Selection of OSH dose. Related to Figure 1, 3 and 4.**
**a.** Experimental scheme: 8 weeks old C57BL/6J male mice were fed for 4 weeks with: chow diet (light blue) and chow diet supplemented with increasing concentrations of OSH (2 - 4 and 8 % respectively gray, brown and blue). **b.** body weight variation expressed as percentage of basal body weight; **c.** fasting blood glucose levels, measured after 4 weeks of feeding with OSH supplemented chow diet; **d.** food intake expressed as food intake in grams per day per mouse; cumulative water intake expressed as water intake in mL per day per mouse; **e.** gut transit time after 1 week OSH treatment; **f.** colon tissue sections of mice fed for 4 weeks with OSH supplemented chow diet stained for ZO-1 (green), CD34 (gray) and DAPI (blue). First line is depicting the three merged channels. Second line is illustrating images of ZO-1 channel alone (gray). Representative images of a single mouse out of 5 mice per group. Scale bar 50 µm; **g.** quantification of ZO-1 fluorescent signal expressed as fold change of control diet. (*$p<0.05$; **$p<0.01$; ***$p<0.001$; **** <0.0001 one-way ANOVA Dunnett's post-test, line at mean with SEM).

## Figure S2

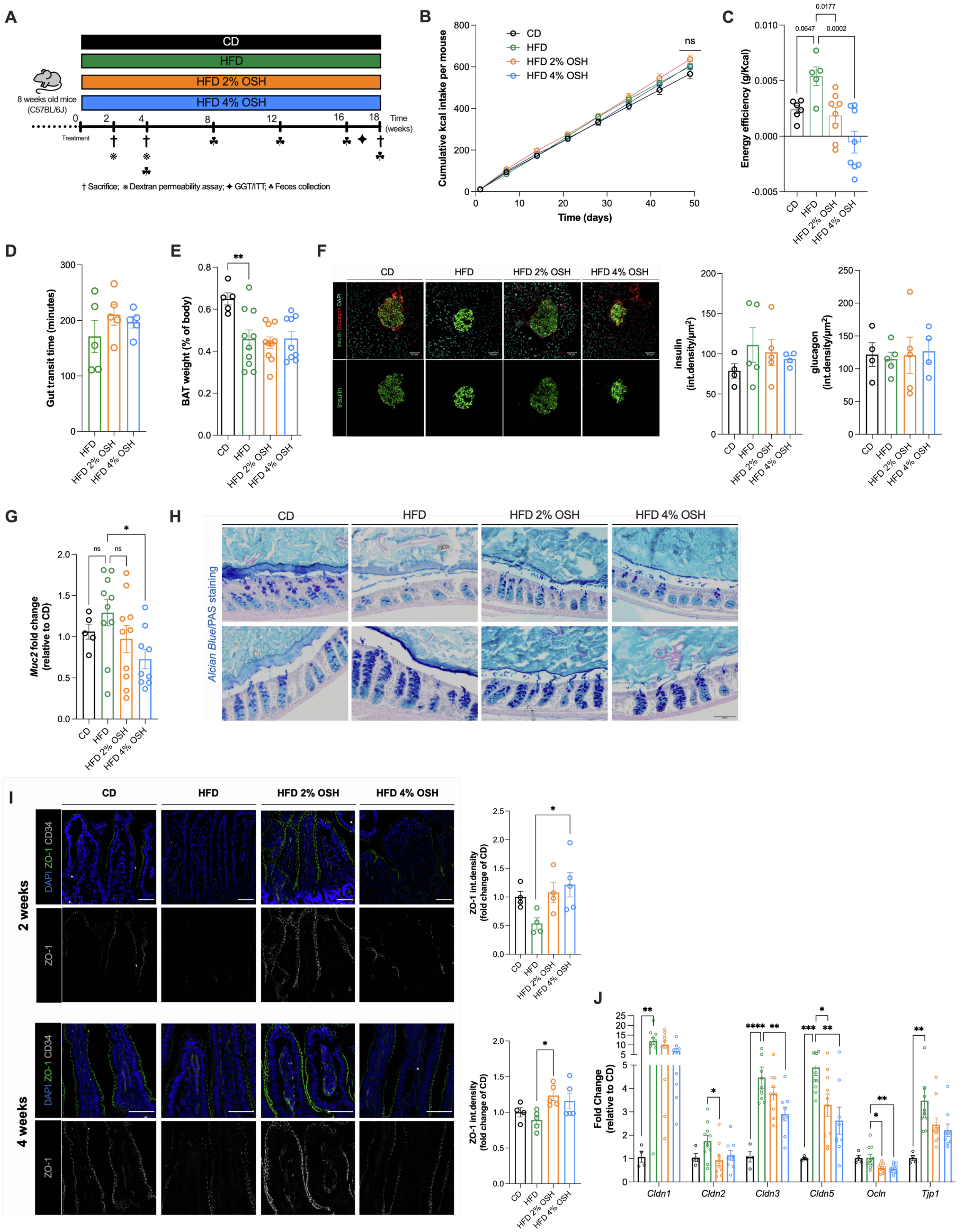


***Supplementary Figure 2.*** **Effects of preventative OSH feeding. Related to Figures 1 and 2.**
C57BL/6J male mice at 8 weeks of age were fed for 18 weeks with: control diet (CD, black) and HFD (green), HFD 2% OSH (orange) and HFD 4% OSH (light blue); after 2- and 4-weeks mice were subjected to 4kDa FITC-Dextran permeability assay and sacrificed to assess intestinal barrier status. Fecal samples were collected after 4,8,12,16 weeks of feeding and at sacrifice for gut microbiota analysis. Glucose Tolerance Test (GTT) and Insulin Tolerance Test (ITT) were performed after 17 weeks of feeding. **b**. Kilocalorie intake, expressed as cumulative kcal intake per mouse, in time (days); **c.** Energy efficiency, calculated as the weight gained over 28 days divided by the calories absorbed over the 28 days for each mouse; **d.** gut transit time after 1 week OSH

treatment; **e.** brown adipose tissue (BAT) weight after 18 weeks of feeding, expressed as percentage of body weight; **f.** pancreatic tissue sections of mice fed for 18 weeks, stained for glucagon (red), insulin (green) and DAPI (cyan). First line is depicting the three merged channels. Second line is illustrating images of insulin channel alone (gray). Representative images of a single mouse out of 4-5 mice per group. Scale bar 50 µm. On the side, quantification of insulin and glucagon fluorescent signal expressed as integrated density per µm$^2$, analyzed using Fiji image software; **g**. Relative *Muc2* gene expression levels in intestinal tissue samples after 18 weeks of OSH treatment expressed as fold change of CD fed mice (n=5 mice per group CD, n=10 mice per group HFD and 2% OSH, n=9 mice per group HFD 4% OSH) (*p<0.05; one-way ANOVA Dunnett's post-test, line at mean with SEM); **h.** Alcian Blue/PAS staining of *Carnoy's* fixed PE colon tissue sections of mice treated for 18 weeks with OSH supplemented diet. Representative images of two mice out of 9/10 animals per group. Scale bar 100 µm; **i.** ileum tissue sections of mice fed for 2 and 4 weeks (lower panel) stained for ZO-1 (green), CD34 (gray) and DAPI (blue). First line is depicting the three merged channels. Second line is illustrating images of ZO-1 channel alone (gray). On the side, respectively, quantification of ZO-1 fluorescent signal expressed as fold change of control diet. Representative images of a single mouse out of 4-5 mice per group. Scale bar 50 µm; **j**. Relative gene expression levels of junctional proteins after 18 weeks of OSH treatment, expressed as fold change of CD fed group (n=4 mice per group CD, n=10 mice per group HFD and 2% OSH, n=9 mice per group HFD 4% OSH) (*p<0.05; **p<0.01; ***p<0.001; **** <0.0001 one-way ANOVA Dunnett's post-test, line at mean with SEM).

## Figure S3

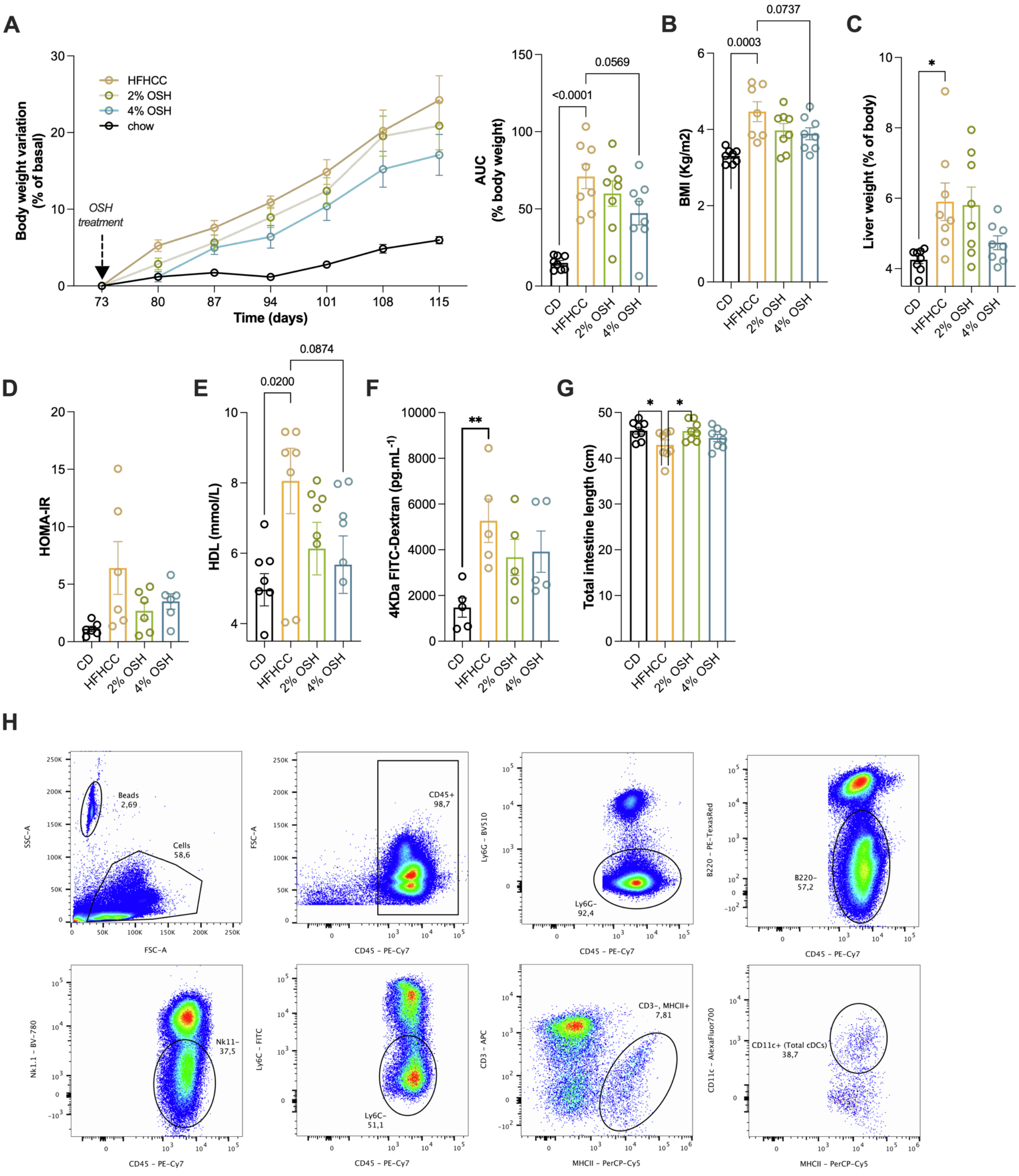

***Supplementary Figure 3*. Effects of therapeutic OSH feeding in NAFLD. Related to Figure 4.**
**a.** Body weight variation in mice therapeutically fed with OSH after 10 weeks of feeding with HFHCC diet. Body weight is expressed as percentage of body weight at day 73 (before OSH administration) and Area Under the Curve (AUC) of body weight variation curve; **b.** Body Mass Index (BMI) after 6 weeks of therapeutic feeding with OSH, calculated as mouse mass in kg/mouse body length in $m^2$; **c.** liver weight expressed as percentage of body weight after 6 weeks of therapeutic feeding with OSH; **d.** HOMA-IR (Homeostatic Model Assessment of Insulin Resistance) values after 6 weeks of therapeutic feeding with OSH; **e.** High-Density Lipoprotein (HDL) serum cholesterol levels and after 6 weeks of therapeutic feeding with OSH; **f.**, Serum 4KDa FITC-Dextran, after 6 weeks of therapeutic feeding with OSH supplemented HFHCC diet; **g.** total intestine length after 18 weeks of feeding. **h.** Gating strategy to study liver myeloid cells by flow cytometry, in mice therapeutically fed with OSH supplemented HFHCC for 6 weeks. (*p<0.05; **p<0.01; ***p<0.001; **** <0.0001 one-way ANOVA Dunnett's post-test, line at mean with SEM).

## Figure S4

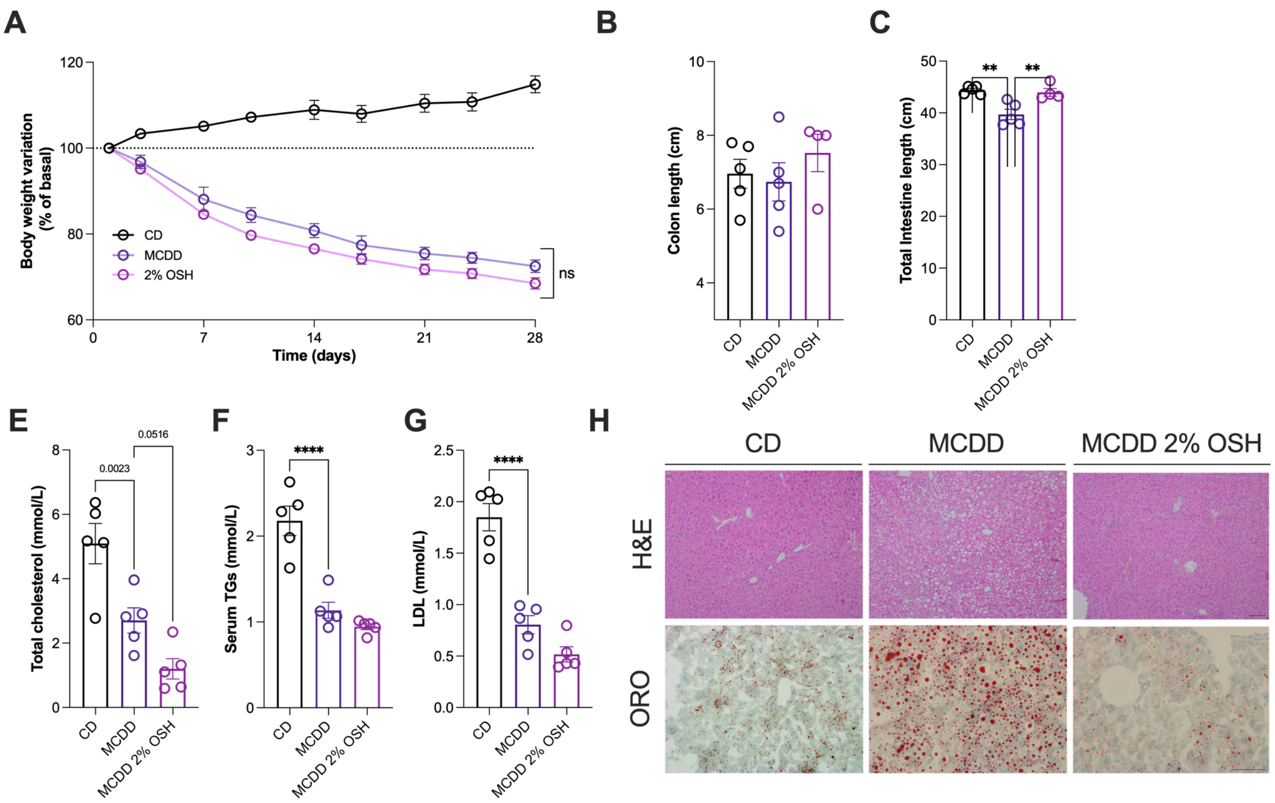


***Supplementary Figure 4*. Effects of preventative OSH administration in a mouse model of NASH. Related to Figure 4.**
C57BL6/J male mice were fed for 4 weeks with MCD diet or MCD diet supplemented with 2 % OSH and control diet (CD). **a.** body weight variation, expressed as percentage of basal body weight; **b.** and **c.** colon length and total intestine length. **e., f.** and **g.** serum total cholesterol, serum total circulating triglycerides and LDL serum cholesterol levels, respectively**,** after 4 weeks of feeding, **h.** liver tissue sections stained for H&E for liver histology (*top line*) and Oil Red O staining for liver triglyceride (*bottom line*), representative images of a single mouse out of 4-5 mice per group, scale bar 100µm. (*p<0.05; **p<0.01; ***p<0.001; **** <0.0001 One-way ANOVA Dunnett's post-hoc test).

## Figure S5

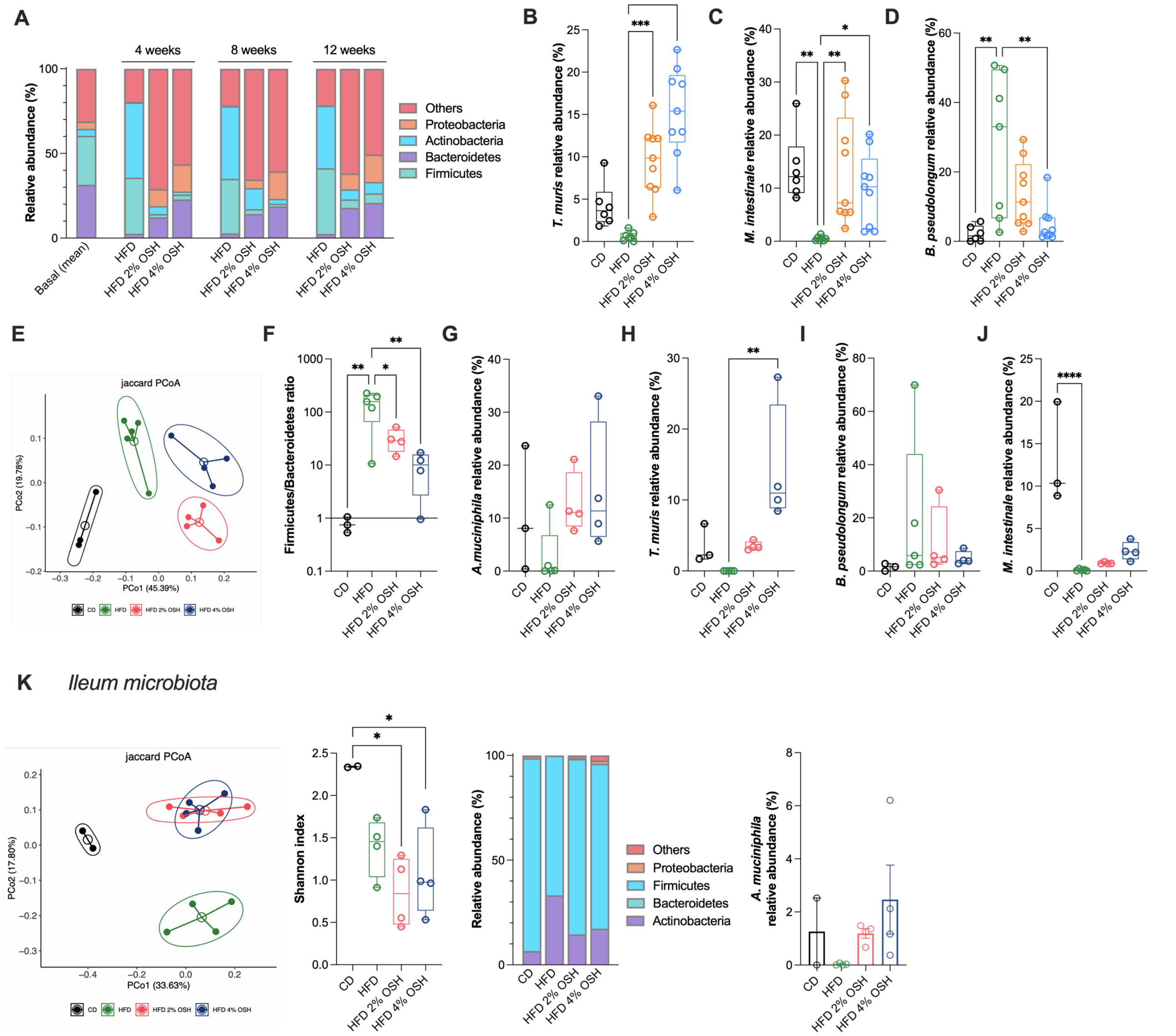


***Supplementary Figure 5*. Gut microbiota modifications induced by preventative and therapeutic OSH administration. Related to Figure 5.**

**a.** Relative abundance of gut microbiota phyla, evolving in time, throughout 12 weeks preventative OSH administration; **b**.**c.** and **d.** *T.muris, M.intestinale and B.pseudolongum* relative abundance in fecal microbiota of mice fed for 18 week with OSH supplemented HFD (*p<0.05; **p<0.01; ***p<0.001; **** <0.0001 One-way ANOVA Dunnett's post-hoc test); **e.** Principal Coordinate Analysis (PCoA), of fecal microbiota from mice therapeutically fed with OSH supplemented diet for 12 weeks - CD (black), HFD (green) and OSH supplemented HFD (2% OSH pink and 4% OSH blue), based on Jaccard index, single dots represent individual mice (PERMANOVA statistical test, p<0,001); **f.** *Firmicutes/Bacteroidetes* ratio in fecal microbiota of mice therapeutically fed with OSH supplemented diet for 12 weeks; **g.**, **h**. **i.** and **j.** *A. muciniphila*, *T.muris, B.pseudolongum* and *M.intestinale* relative abundance in fecal microbiota of mice therapeutically fed with OSH supplemented diet for 12 weeks (*p<0.05; **p<0.01; ***p<0.001; **** <0.0001 One-way ANOVA Dunnett's post-hoc test); **k.** Analysis of ileum content microbiota of mice therapeutically fed with OSH supplemented diet for 12 weeks, Principal Coordinate Analysis (PCoA), based on Jaccard index, single dots represent pooled samples from individual mice (5 pooled samples for each dot for CD; 3-1-2 and 3 samples for HFD; 3-3-2 and 2 samples for 2% OSH and 3-3-3 and 1 samples for 4% OSH); Shannon's diversity index of ileum microbiota (*p<0.05; One-way ANOVA Dunnett's post hoc test); Relative abundance of detected fecal bacteria phyla expressed as percentage of total; *A.muciniphila* relative abundance in ileum content microbiota of mice therapeutically fed with OSH supplemented diet for 12 weeks.

## Figure S6

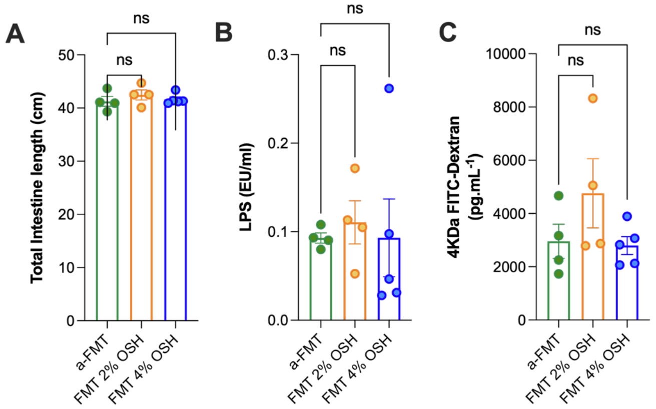


***Supplementary Figure 6*. FMT recipients' metabolic parameters. Related to Figure 7.**
**a.** Total intestine length of recipient mice receiving FMT every other day for 4 weeks; **b.** Circulating LPS levels of recipient mice receiving FMT every other day for 4 weeks; **c.** Plasma FITC-Dextran (4kDa) levels of recipient mice receiving FMT every other day for 4 weeks (One-way ANOVA Dunnett's post-hoc test).

***Supplementary Table 1*. Animal diet composition. Related to STAR METHODS Experimental Model and Study Participant section.**

| | Low Fat control | Standard Chow control | HFD | MCDD | HFHCC |
|---|---|---|---|---|---|
| **Code** | TD.86489 | 4RF21 | TD.06415 | TD.90262 | 5TJT |
| **Supplier** | ENVIGO | MUCEDOLA | ENVIGO | ENVIGO | TESTDIET |
| **Purified diet** | Yes | No | Yes | Yes | Yes |
| **Total energy (Kcal/g)** | 3.7 | 3.9 | 4.6 | 4.1 | 4.5 |
| **Protein (%kcal from)** | 20.0 | 18.5 | 19.0 | 14.6 | 16.1 |
| **Carbohydrate (%kcal from)** | 67.3 | 53.5 | 36.2 | 63.2 | 44.2 |
| **Fat (%kcal from)** | 12.8 | 6.0 | 44.8 | 22.1 | 39.7 |
| **Ingredients (g/Kg)** | | | | | |
| **Casein** | 207 | | 245 | | 196 |
| **Sucrose** | 321 | | 200 | 455 | 78 |
| **Starch** | 320 | | 85 | 200 | 309 |
| **Malto / dextrin** | | | 115 | | 103 |
| **Lard** | | | 195 | | 60 |
| **Oil (soybean or corn)** | 30 | | 30 | 100 | 18 |
| **Crisco** | | | | | 60 |
| **Milk Fat** | | | | | 60 |
| **Cellulose** | 50 | | 58 | 50 | 25 |

***Supplementary Table 2*. Primer sequences. Related to STAR METHODS Quantification of DNA and mRNA by Real-Time PCR section.**

| **Oligonucleotides** | | | |
|---|---|---|---|
| **Gene** | **Sequence Fwd** | **Sequence Rev** | **Source** |
| *Slc27a4* | ACTGTTCTCCAAGCTAGTGCT | GATGAAGACCCGGATGAAACG | Sigma |
| *Ffar2* | CCTCGGTCCAGTGCTGTG | GCCCTGGGACTCGTCAAG | Sigma |
| *Cd36* | AGATGACGTGGCAAAGAACAG | CCTTGGCTAGATAACGAACTCTG | Sigma |
| *Fabp6* | CTTCCAGGAGACGTGATTGAAA | AACTTGTTGCTCATAATGTTGCC | Sigma |
| *Ppara* | AACATCGAGTGTCGAATATGTGG | CCGAATAGTTCGCCGAAAGAA | Sigma |
| *Pparg* | GGAAGACCACTCGCATTCCTT | GTAATCAGCAACCATTGGGTCA | Sigma |
| *Cpt1a* | CTCCGCCTGAGCCATGAAG | CACCAGTGATGATGCCATTCT | Sigma |
| *Mlxipl* | AGATGGAGAACCGACGTATCA | ACTGAGCGTGCTGACAAGTC | Sigma |
| *Fasn* | GGAGGTGGTGATAGCCGGTAT | TGGGTAATCCATAGAGCCCAG | Sigma |
| *Cldn1* | GAGGGACTGTGGATGTCCTG | ATGCCAATTACCATCAAGGC | Sigma |
| *Cldn2* | TTAGCCCTGACCGAGAAAGA | AAAGGACCTCTCTGGTGCTG | Sigma |
| *Cldn3* | GAGATGGGAGCTGGGTTGT | GGATCTTGGTGGGTGCATAC | Sigma |
| *Cldn5* | AGCTGGTGGCACTCTTTGTT | GCACCGTCGGATCATAGAAC | Sigma |
| *Ocln* | CCTCCAATGGCAAAGTGAAT | AGGAATCTCCTGGGCCACT | Sigma |
| *Tjp1* | CGCGGAGAGAGACAAGATGT | CCTGTGAAGCGTCAGTGTGT | Sigma |
| *16S rRNA* | ACTCCTACGGGAGGCAGCAGT | ATTACCGCGGCTGCTGGC | Sigma |
| *16S rRNA_Amuciniphila* | CAGCACGTGAAGGTGGGGAC | CCTTGCGGTTGGCTTCAGAT | Sigma |
| *Muc2* | CCTGAAGGCACCACAAAGT | TTGCAGTCAAACTCAAAGT | Sigma |
