# Supplementary material for: Biomimetic superabsorbent hydrogel acts as a gut protective dynamic exoskeleton improving metabolic parameters and expanding A. muciniphila
Source: Cell Rep Med. 2023 Oct 17;4(10):101235. doi: 10.1016/j.xcrm.2023.101235 (PMC10591066; doi:10.1016/j.xcrm.2023.101235)
Supplement: Document S2. Article plus supplemental information [file mmc2.pdf]

Cell Reports
Medicine

Article

Biomimetic superabsorbent hydrogel acts as a gut protective dynamic exoskeleton improving metabolic parameters and expanding *A. muciniphila*

Graphical abstract

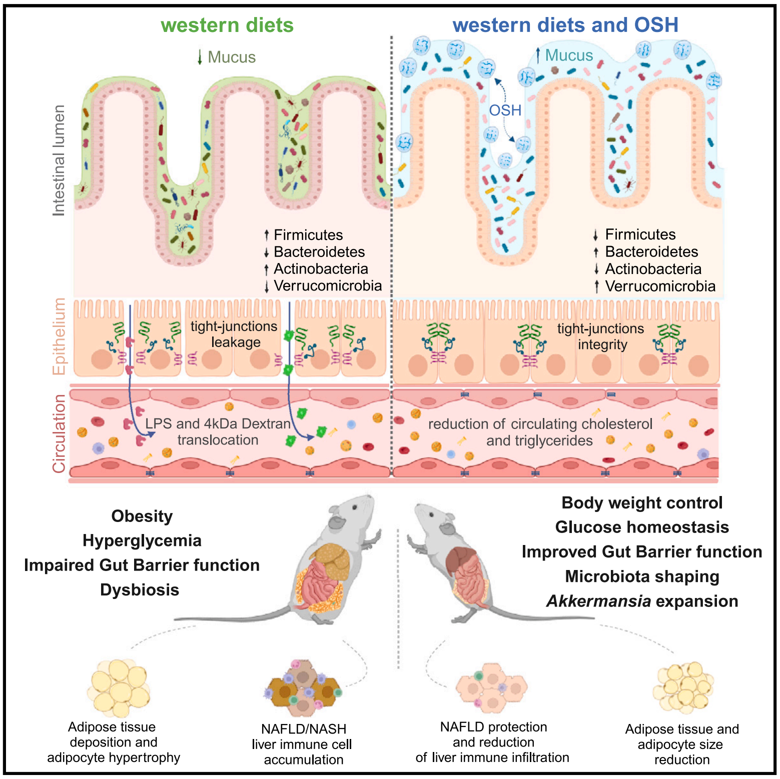

Highlights

- Oral superabsorbent hydrogel (OSH) improves metabolic parameters in mice
- Two mechanisms are involved: protects intestinal barriers and shapes the gut microbiota
- Chemical and physical properties of OSH foster *Akkermansia muciniphila* growth

Authors

Alessandra Silvestri, Antonio Gil-Gomez, Milena Vitale, ..., Giuseppe Penna, Alessandro Sannino, Maria Rescigno

Correspondence

maria.rescigno@hunimed.eu

In brief

In brief, Silvestri et al. evaluate the therapeutic effects of a superabsorbent hydrogel as a non-systemic mechanobiological approach to treat metabolic syndrome and its comorbidities. Results imply that this device improves intestinal barrier protection while inducing changes in gut microbiota composition, specifically fostering *Akkermansia muciniphila* expansion.

Silvestri et al., 2023, Cell Reports Medicine *4*, 101235
October 17, 2023 © 2023 The Author(s).
https://doi.org/10.1016/j.xcrm.2023.101235

CellPress

Cell Reports Medicine

CellPress
OPEN ACCESS

Article

Biomimetic superabsorbent hydrogel acts as a gut protective dynamic exoskeleton improving metabolic parameters and expanding *A. muciniphila*

Alessandra Silvestri,[1,8] Antonio Gil-Gomez,[1,2,3,8] Milena Vitale,[1] Daniele Braga,[1] Christian Demitri,[4,5] Paola Brescia,[1,6] Marta Madaghiele,[4,5] Ilaria Spadoni,[1,6] Bryan Jones,[7] Giulia Fornasa,[1] Juliette Mouries,[1] Sara Carloni,[1,6] Michela Lizier,[1] Manuel Romero-Gomez,[2,3] Giuseppe Penna,[1] Alessandro Sannino,[4,7] and Maria Rescigno[1,6,9,*]

[1]IRCCS Humanitas Research Hospital, Via Manzoni 56, 20089 Rozzano, Milan, Italy
[2]Instituto de Biomedicina de Sevilla (IBiS), Hospital Universitario Virgen del Rocío/CSIC/Universidad de Sevilla, 41013 Seville, Spain
[3]Centro de Investigación Biomédica en Red de Enfermedades Hepáticas y Digestivas (CIBERehd), 28029 Madrid, Spain
[4]Department of Engineering for Innovation, University of Salento, Via per Monteroni, 73100 Lecce, Italy
[5]Gelesis, 73021 Calimera, Lecce, Italy
[6]Department of Biomedical Sciences, Humanitas University, Via Rita Levi Montalcini 4, 20072 Pieve Emanuele, Milan, Italy
[7]Gelesis, Boston, MA 02116, USA
[8]These authors contributed equally
[9]Lead contact
*Correspondence: maria.rescigno@hunimed.eu
https://doi.org/10.1016/j.xcrm.2023.101235

SUMMARY

The rising prevalence of obesity and metabolic disorders worldwide highlights the urgent need to find new long-term and clinically meaningful weight-loss therapies. Here, we evaluate the therapeutic potential and the mechanism of action of a biomimetic cellulose-based oral superabsorbent hydrogel (OSH). Treatment with OSH exerts effects on intestinal tissue and gut microbiota composition, functioning like a protective dynamic exoskeleton. It protects from gut barrier permeability disruption and induces rapid and consistent changes in the gut microbiota composition, specifically fostering *Akkermansia muciniphila* expansion. The mechanobiological, physical, and chemical structures of the gel are required for *A. muciniphila* growth. OSH treatment induces weight loss and reduces fat accumulation, in both preventative and therapeutic settings. OSH usage also prevents liver steatosis, immune infiltration, and fibrosis, limiting the progression of non-alcoholic fatty liver disease. Our work shows the potential of using OSH as a non-systemic mechanobiological approach to treat metabolic syndrome and its comorbidities.

INTRODUCTION

Metabolic syndrome (MetS) is defined as the accumulation of various symptoms that increase the risk of cardiovascular disease, diabetes mellitus, non-alcoholic fatty liver disease (NAFLD), and all-cause mortality. It is caused by a long-term imbalance in energy homeostasis that leads to the formation of fat depots and weight gain due to a combination of poor diet, sedentary lifestyle, and genetic predisposition.[1] The crux of MetS is an impairment in glucose, lipid, and protein metabolism producing a buildup and dysfunction of adipose tissue that leads to insulin resistance and proinflammatory cytokine secretion. This could trigger a chronic, low-grade inflammatory state, aggravated by the leakage of proinflammatory gut-derived molecules into the systemic circulation due to impaired gut barrier integrity.[2] MetS is associated with a spectrum of liver damage causing steatosis that can progress to steatohepatitis and fibrosis. Indeed, the incidences of diabetes, cardiovascular diseases, and NAFLD have increased dramatically in the past decades, paralleling the rising prevalence of obesity.[3] It is expected that, by 2030, 80% of adults in the United States will be overweight or obese, which is associated with both an increased risk of all-cause mortality and a reduced quality of life, resulting in a social and economic burden with increasingly alarming incidence.[1,4,5]

The management of obesity and its associated metabolic disorders is currently based on dietary or lifestyle approaches and pharmacological and surgical interventions, mostly focused on achieving weight loss. To date, the majority of the approved therapeutics have demonstrated modest efficacy (3%–8% weight loss), and their safety and/or tolerability profiles may limit their long-term utilization. Newer weight-loss medications have demonstrated higher efficacy by mostly affecting the central nervous system[6]; however, recent data suggest that MetS may initiate with deregulation of intestinal homeostasis and increased gut permeability.[7] There is, hence, an urgent need to find new therapies that could result in clinically meaningful weight loss by restoring gut homeostasis, so as to prevent the onset of or ameliorate the associated MetS.

© 2023 The Author(s).
This is an open access article under the CC BY license (http://creativecommons.org/licenses/by/4.0/).

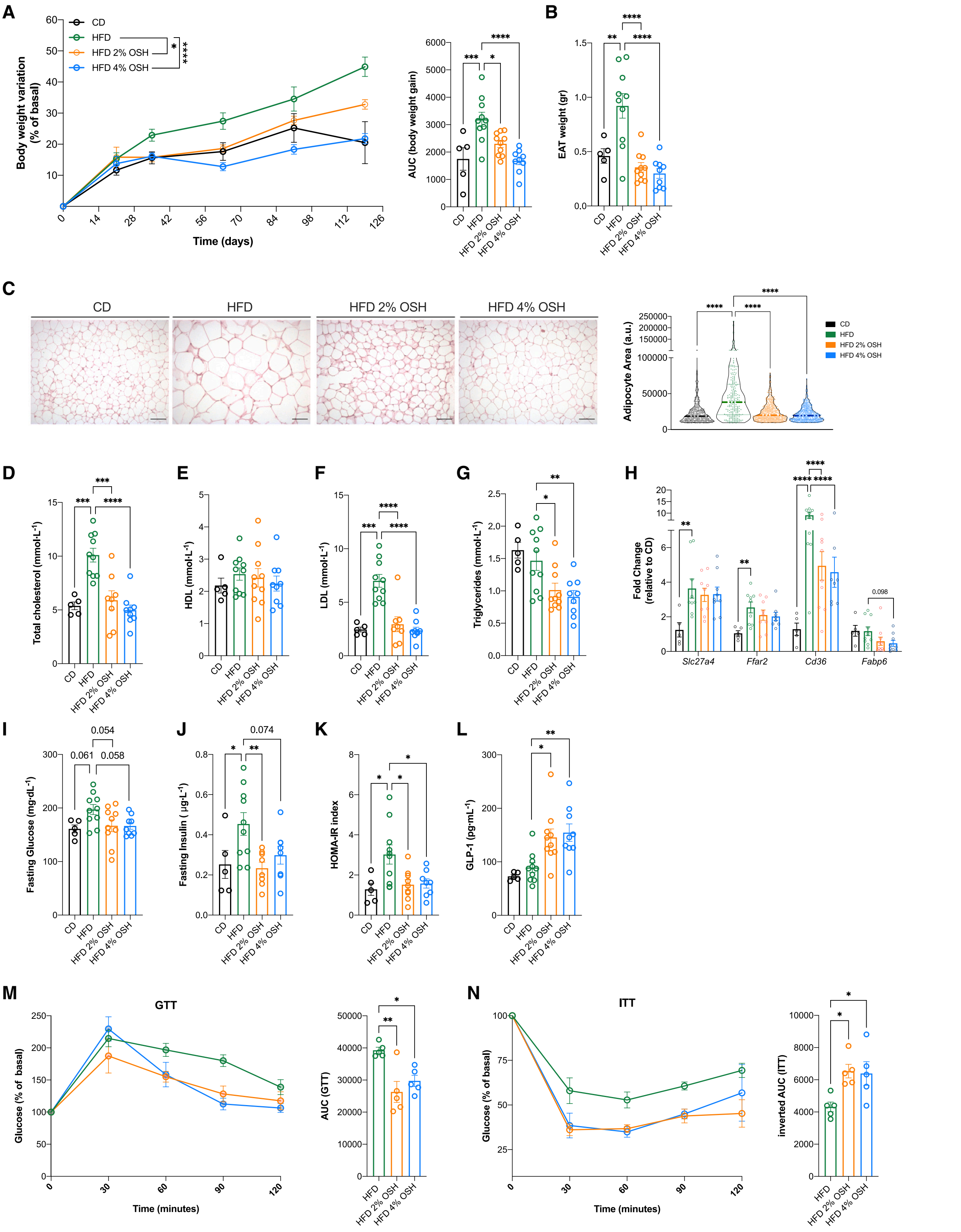

(legend on next page)

Alterations in gut microbiota composition play a key role in the development of obesity,[8] MetS,[9] and NAFLD,[10] although the mechanisms by which gut microbiota promote the metabolic alterations are still not understood. Several reports have linked obesity-associated gut microbiota to insulin resistance through alterations in lipopolysaccharide (LPS)[11] together with increased energy harvest by obesity-associated gut microbiota.[12] The microbiota in obesity is characterized by a decrease in taxa belonging to Bacteroidetes and Verrucomicrobia with a proportional increase in members of the Firmicutes and Actinobacteria phyla.[8] In addition, recent findings unveiled an association between *Akkermansia muciniphila* (Verrucomicrobia) abundance with a healthier metabolic state and an improvement in lipid and carbohydrate metabolism and body fat distribution, with a decrease in waist circumference.[13] Therefore, modulation of microbiota can be an effective means of weight control in obesity and metabolic disorders.

Gelesis's oral superabsorbent hydrogels (OSHs) are cross-linked cellulose-based materials specifically designed to provide a new non-systemic approach for treating obesity.[14] We recently showed[15] that OSHs, which are entirely based on generally recognized as safe (GRAS) building blocks, are engineered to mimic basic compositional and mechanical features of ingested raw vegetables, making them unique among the several available hydrogels. Taken as capsules prior to a meal, OSH products expand by hydration in the gastrointestinal system to reduce the caloric density of the meal and to increase the volume and the elasticity of the ingested foods throughout the digestive system, in a manner similar to that of raw vegetables. This biomimetic approach holds promise for safe (long-term use) and effective weight management in the treatment of obesity.

While Plenity (GS100) has been cleared in the US and Europe as a useful aid for weight management, it is yet unclear whether and how the intestinal transit of given hydrogel formulations, maintaining structural integrity with known volume and a specific range of elasticity during small intestinal transit, may affect gut health and induce metabolic changes.[16] It is thus reasonable that the further interaction of the material with the intestinal walls, in addition to the stomach, may increase its therapeutic efficacy compared with other OSH products.

In this study, we aimed to investigate the efficacy and mechanism of action of a new experimental OSH formulation (GS200)[15] with an elastic profile higher than that of functional fibers and of GS100, but similar to that of raw vegetables, in preclinical murine models of diet-induced obesity, MetS, and non-alcoholic steatohepatitis (NASH), studying the potential involvement of different systems and organs in an integrated manner. Here, we demonstrate that, by targeting the gut-liver axis, OSH is able to prevent and reverse MetS, limiting NAFLD progression. This is achieved by direct effects on the intestinal barrier and the gut microbiota, based on OSH mechanobiological properties.

RESULTS

OSH administration prevents diet-induced obesity and metabolic syndrome

To investigate the effects of the superabsorbent hydrogel, we induced obesity and metabolic alterations in mice through a dietary regimen rich in saturated fats from animal sources (45% kcal from fat; HFD) with or without the addition of 2% or 4% OSH for up to 18 weeks. The selection of the doses was based on a previous experiment that showed their safety and mild/intermediate efficacy in lowering body weight or controlling glucose levels in standard-diet-fed mice (Figures S1A–S1C). After 18 weeks, the HFD led to the development of obesity, whereas mice fed an HFD with either 2% or 4% OSH showed a dose-dependent reduction in body weight gain compared with the HFD alone (35.4 ± 1.3 g vs. 30.3 ± 0.8 g vs. 30.1 ± 0.6 g; $p < 0.001$) (Figure 1A). In agreement with a recent human proof-of-concept study (NCT04257123),[16] we did not observe significant changes in food intake (Figures S1D and S2B), gut transit time (Figures S1E and S2D), or energy absorption (data not shown); nevertheless, administration of the hydrogel was associated with a decrease in energy efficiency (Figures S2A–S2C). In addition, it was able to fully prevent adipose tissue hypertrophy and remodeling induced by HFD, as shown by a lower epididymal adipose tissue (EAT) weight ($p < 0.001$) (Figure 1B) with a significant drop in adipocyte size (Figure 1C). We did not observe any significant change in brown adipose tissue (BAT) deposition in treated animals (Figure S2E).

As expected, HFD-fed mice presented with hypercholesterolemia, mostly due to the increase in serum LDL particles ($p < 0.005$ compared with control diet [CD]), whereas mice receiving the hydrogel were protected from the increase in total cholesterol levels

Figure 1. OSH administration prevents obesity and metabolic syndrome in mice

C57BL/6J male mice were fed for 18 weeks with HFD or HFD supplemented with 2% or 4% OSH or control diet (CD).

(A) Body weight variation as percentage of basal; area under the curve (AUC) of body weight variation.

(B) Epididymal adipose tissue (EAT) weight.

(C) H&E staining of EAT tissue sections, scale bar, 100 μm; epididymal adipocyte area distribution, line at median; 75–100 cells per field per mouse; five mice per group were analyzed.

(D) Serum total cholesterol levels.

(E–G) High-density lipoprotein (HDL) and low-density lipoprotein (LDL) serum cholesterol levels and total circulating triglyceride levels, respectively.

(H) Relative gene expression levels of the lipid and fatty acid transporters *Slc27a4*, *Ffar2*, *Cd36*, and *Fabp6* in the ileum at 18 weeks, expressed as fold change of CD-fed group (n = 5 CD; n = 9 HFD and 2%–4% OSH).

(I and J) Fasting blood glucose and insulin levels (n = 5 CD, n = 10 HFD, n = 8 2%–4% OSH).

(K) HOMA-IR values.

(L) Circulating GLP-1 levels.

(M) Intraperitoneal GTT expressed as a percentage of basal performed after 17 weeks of feeding (n = 5 mice per group); AUC of GTT.

(N) Intraperitoneal ITT as a percentage of basal performed after 17 weeks of feeding (n = 5 mice per group); inverted AUC of ITT (*$p < 0.05$; **$p < 0.01$; ***$p < 0.001$ one-way ANOVA Dunnett post-test, line at mean with SEM).

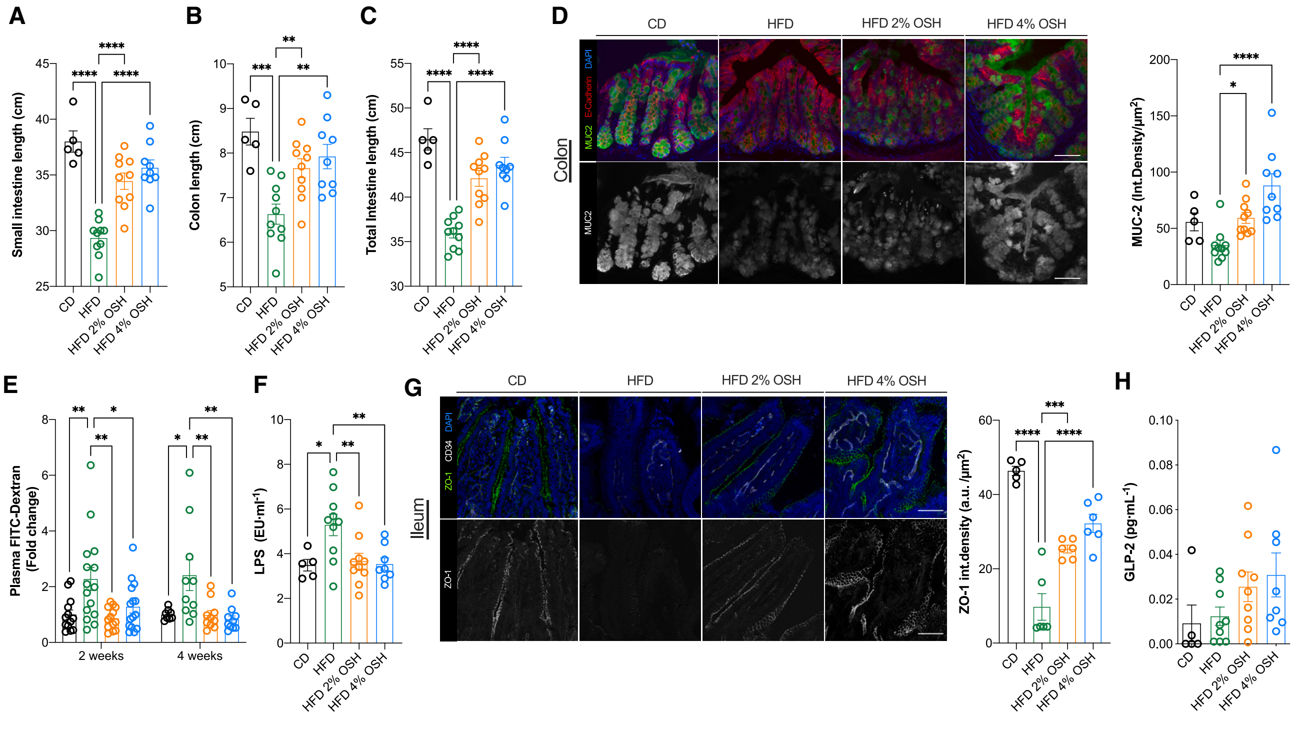

Figure 2. OSH administration protects intestinal barrier from HFD-induced alterations

(A–C) Small intestine, colon, and total intestine length, respectively, after 18 weeks of feeding.

(D) Colon tissue sections of mice fed for 18 weeks, stained for Muc-2 (in green), E-cadherin (in red), and DAPI, marker for nuclei (in blue). Scale bar, 50 µm.

(E) Plasma FITC-dextran (4 kDa) levels after 2 and 4 weeks of feeding, expressed as fold change of CD (n = 15 for 2 weeks testing, n = 10 for 4 weeks testing).

(F) Circulating LPS levels after 18 weeks of feeding.

(G) Ileum tissue sections stained for ZO-1, marker of tight junctions (in green); CD34, marker of vessels (in gray); and DAPI, marker for nuclei (in blue). Scale bar, 50 µm.

(H) GLP-2 serum levels. Fluorescent signals are expressed as integrated density/μm^2 and analyzed using Fiji image software (*$p < 0.05$; **$p < 0.01$; ***$p < 0.001$; ****$p < 0.0001$; one-way ANOVA Dunnett's post-test, line at mean with SEM; n = 5 [CD], n = 10 mice per group).

by impeding serum triglyceride and LDL accumulation (Figures 1D–1G). This effect could be achieved by a regulation of liver lipid metabolism or a decrease in intestinal absorption. Indeed, mice fed an HFD had increased expression of the ileal fatty acid transporters *Slc27a4*, *Ffar2*, and *Cd36*, while mice receiving OSH showed lower intestinal *Cd36* and *Fabp6* expression levels, suggesting a reduction in fatty acid uptake (Figure 1H).

Preventative administration of OSH also exerted an effect in controlling metabolic alterations in glucose homeostasis and insulin sensitivity, with mice displaying lower levels of fasting glycemia and insulinemia, mirrored by a lower homeostatic model assessment of insulin resistance (HOMA-IR) index (Figures 1I–1K). In these mice, we also observed a hydrogel-dependent increase in GLP-1 serum levels ($p = 0.011$ for 2%, $p = 0.004$ for 4%) (Figure 1L). Interestingly, mice fed with HFD plus 2% or 4% OSH had a better response to both insulin and glucose loads in the insulin tolerance test (ITT) and glucose tolerance test (GTT) (Figures 1M and 1N). We did not observe significant structural changes in pancreatic islets after HFD feeding, meaning that hyperinsulinemia was supported by β cell hyperfunction, but ineffective β cell compensation led to impaired glucose tolerance (Figure S2F). Altogether, these data support the ability of OSH to improve glucose tolerance and prevent insulin resistance development, a hallmark of MetS, and an association with GLP-1 increase.

OSH preserves the gut barrier integrity, protecting from HFD-induced "leaky gut"

After 18 weeks of feeding, mice fed an HFD showed a significant reduction in the small intestine and colon length, an established sign of gut impairment likely promoted by the induction of a low-grade inflammatory state due to the toxicity caused by HFD feeding.[17,18] Remarkably, OSH treatment prevented the intestinal shortening at both doses (Figures 2A–2C). We analyzed the mucus barrier and found that OSH prevented the disruption induced by the diet, stimulating the production of mucin-2, a key component of the colonic mucus barrier (Figures 2D, S2G, and S2H). The increased mucus layer not only would protect from colonization and/or translocation of microbial pathogens but also could contribute to the mutualism between host and certain bacteria.[19]

We previously reported that intestinal barrier disruption caused by HFD occurs early in the development of MetS and results in the translocation of bacteria and their subproducts.[7] These molecules (i.e., microbe-associated molecular patterns [MAMPs]; damage-associated molecular patterns [DAMPs]) reach distant

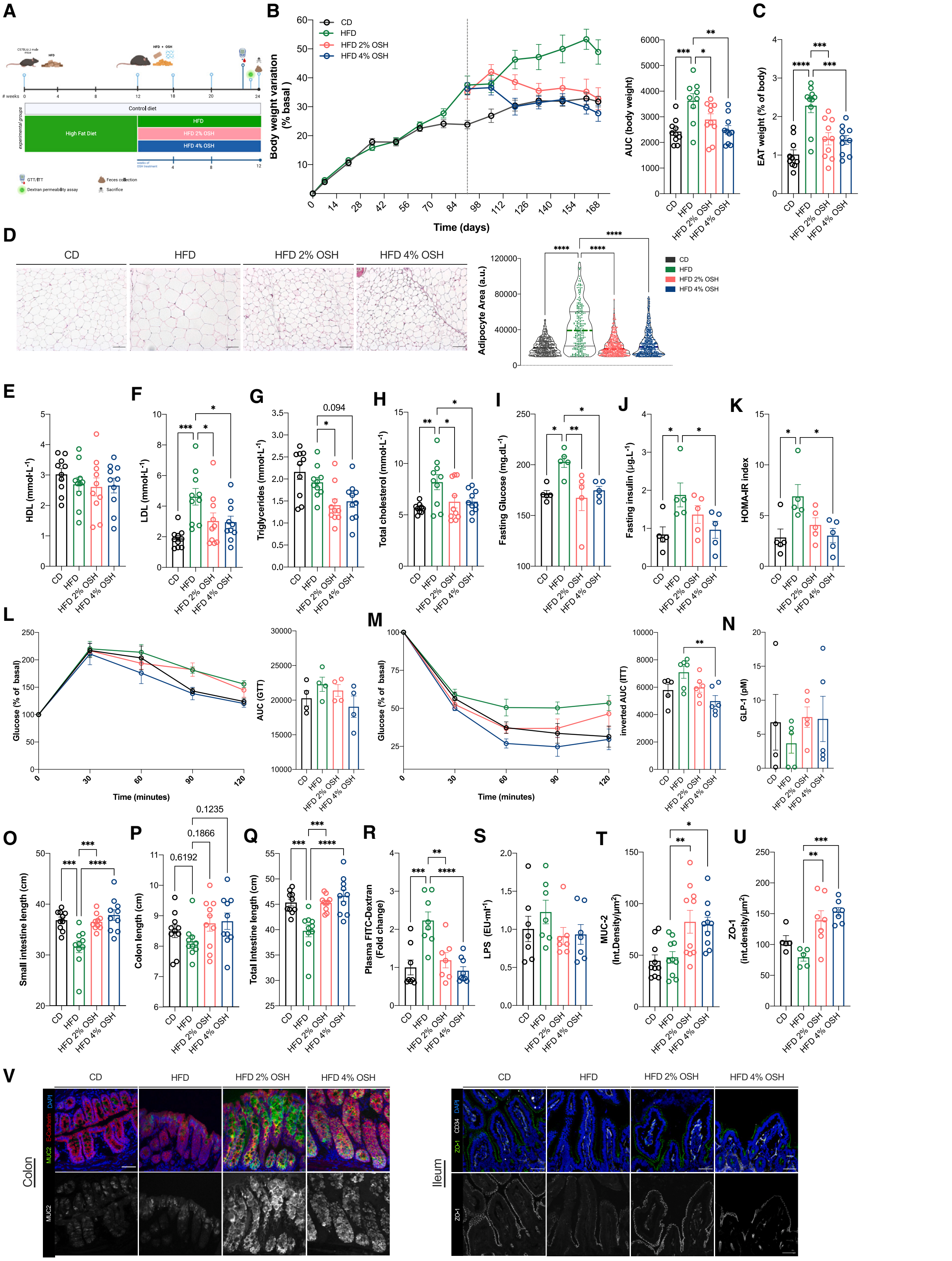

(legend on next page)

organs, inducing low-grade inflammatory processes, and play a role in the progression of metabolic disorders.[7] Here, we confirm the functional alteration in intestinal permeability in as soon as 2–4 weeks of HFD feeding, indicated by higher diffusion of 4 kDa fluorescein isothiocyanate (FITC)-dextran into the bloodstream. OSH treatment conferred protection from increased gut permeability at both concentrations (Figure 2E). This effect was also paralleled by protection against metabolic endotoxemia, evaluated by plasma LPS levels (Figure 2F).

After observing this functional rescue, we hypothesized that epithelial tight junctions, the primary determinants of paracellular permeability, could also be involved. In line with this, ZO-1 protein expression was inversely correlated with the FITC-dextran assay and plasma LPS, consistent with a protective activity of the hydrogel against epithelial barrier disruption, not only in the short term (Figure S2I), but also after 18 weeks of HFD (Figure 2G). Curiously, we found an opposite trend in the expression levels of tight-junction genes (Figure S2J), probably as a means to counteract the downregulation at the protein level; still, the overexpression of *Tjp1*, *Cldn2*, *Cldn3*, and *Cldn5* tended to normalize upon treatment. A discordance between protein and mRNA expression for stable proteins in a perturbed state in the ileum has been recently described.[20] We also observed a non-statistically significant increase compared with HFD alone of 2.07- and 2.51-fold (for 2% and 4%, respectively) for GLP-2 (Figure 2H), an intestinal hormone with trophic and direct protective functions at the intestinal epithelium,[21] which could explain the improvement in gut barrier integrity. Together, these data demonstrate that the hydrogel is able to prevent HFD-induced alterations in the intestinal barrier, preventing epithelial tight-junction loss and increased functional permeability.

OSH reverses metabolic impairment in DIO C57BL/6J mice

We then decided to evaluate the therapeutic potential of the hydrogel for the treatment of MetS. To this end, we first induced the metabolic alterations in C57BL/6J male mice through HFD feeding for 12 weeks. At the end of the induction phase, mice were randomly allocated into HFD, HFD +2% OSH, and HFD +4% OSH groups and monitored for 12 additional weeks (therapeutic phase) (Figure 3A). Mice receiving OSH started to lose weight from the beginning of the treatment, reaching levels comparable to those of the CD group after 4 weeks. After 12 weeks of treatment, mice lost 2.2% and 8.5% of weight (2% and 4%, respectively) from baseline, while mice on HFD alone gained 11.4% of weight (Figure 3B). This was accompanied by a significant reduction in fat accumulation in the EAT, reflected by a decrease in the size of adipocytes (hypertrophy) (Figures 3C and 3D). In this setting, with established hyperlipidemia before treatment, we confirmed the cholesterol-lowering capacity of the hydrogel, observing that treated mice exhibited a decline in serum cholesterol, mostly at the expense of LDL (Figures 3E–3H).

The therapeutic administration of OSH also reversed the impairment in glucose metabolism induced by the diet, returning glycemia and insulinemia levels close to those of control animals, in a dose-dependent fashion (Figures 3I–3K). Consequently, it decreased insulin resistance (HOMA-IR: 2% $p = 0.089$, 4% $p = 0.017$ vs. HFD) (Figure 3K). The responses to both GTT and ITT were improved, demonstrating that mice treated with OSH had better glucose tolerance and insulin sensitivity, particularly after 4% OSH administration (Figures 3L and 3M). Further, GLP-1 levels were 2.1- and 2.0-fold higher in mice with 2% and 4% hydrogel compared with HFD, although statistical significance was not reached (Figure 3N).

We observed a recovery in HFD-induced intestinal damage with the treatment. The shortening of the intestine (more evident at the level of the small intestine) was completely recovered (Figures 3O–3Q). FITC-dextran translocation in the bloodstream after gut permeability challenge demonstrated a functional recovery of the barrier by OSH administration, also reflected by the reduced amount of plasma LPS (p = ns) (Figures 3R and

Figure 3. OSH therapeutic administration over 12 weeks reverses obesity and metabolic alterations induced by HFD feeding
(A) Experimental design for therapeutic administration of OSH.
(B) Body weight variation expressed in percentage of basal; area under the curve.
(C) EAT weight.
(D) H&E staining of EAT formalin-fixed paraffin-embedded tissue sections, scale bar, 100 μm. Epididymal adipocyte area distribution, line at median; 75–100 cells per field per mouse, five mice per group were analyzed.
(E–H) HDL and LDL serum levels, total circulating triglycerides, and total cholesterol.
(I and J) Fasting blood glucose and insulin levels (n = 5 mice per group).
(K) HOMA-IR values (n = 5 mice per group.
(L) Intraperitoneal GTT as a percentage of basal performed after 23 weeks of feeding (n = 5 mice per group); AUC of GTT.
(M) Intraperitoneal ITT (n = 5 per group); inverted AUC of ITT.
(N) Circulating GLP-1 levels.
(O–Q) Small intestine, colon, and total intestine length.
(R) Plasma FITC-dextran (4 kDa) levels (n = 8 CD, n = 8 HFD, n = 7 2% OSH, n = 9 4% OSH; outliers were calculated using GraphPad outlier calculator and excluded from analysis).
(S) Circulating LPS levels after 12 weeks of OSH treatment (n = 7 mice per group).
(T) Quantification of Muc-2 fluorescent signal expressed as integrated density/μm^2, performed using Fiji image software.
(U) Quantification of ZO-1 fluorescent signal expressed as integrated density/μm^2, performed using Fiji image software (n = 5 CD and HFD, n = 7 for 2% and 4% OSH).
(V) Colon (left) sections stained for Muc-2 (in green), E-cadherin (in red), and DAPI (in blue). Ileum tissue (right) sections stained for ZO-1 (in green); CD34, a marker of vessels (in gray); and DAPI (in blue). Representative images of a single mouse of five for CD and HFD and of seven for 2% and 4% OSH groups. Scale bar, 50 μm (*$p < 0.05$; **$p < 0.01$; ***$p < 0.001$; ****$p < 0.0001$; one-way ANOVA Dunnett's post-test, line at mean with SEM; number of mice per group n = 10 HFD in all graphs if not specified differently).

CellPress
OPEN ACCESS

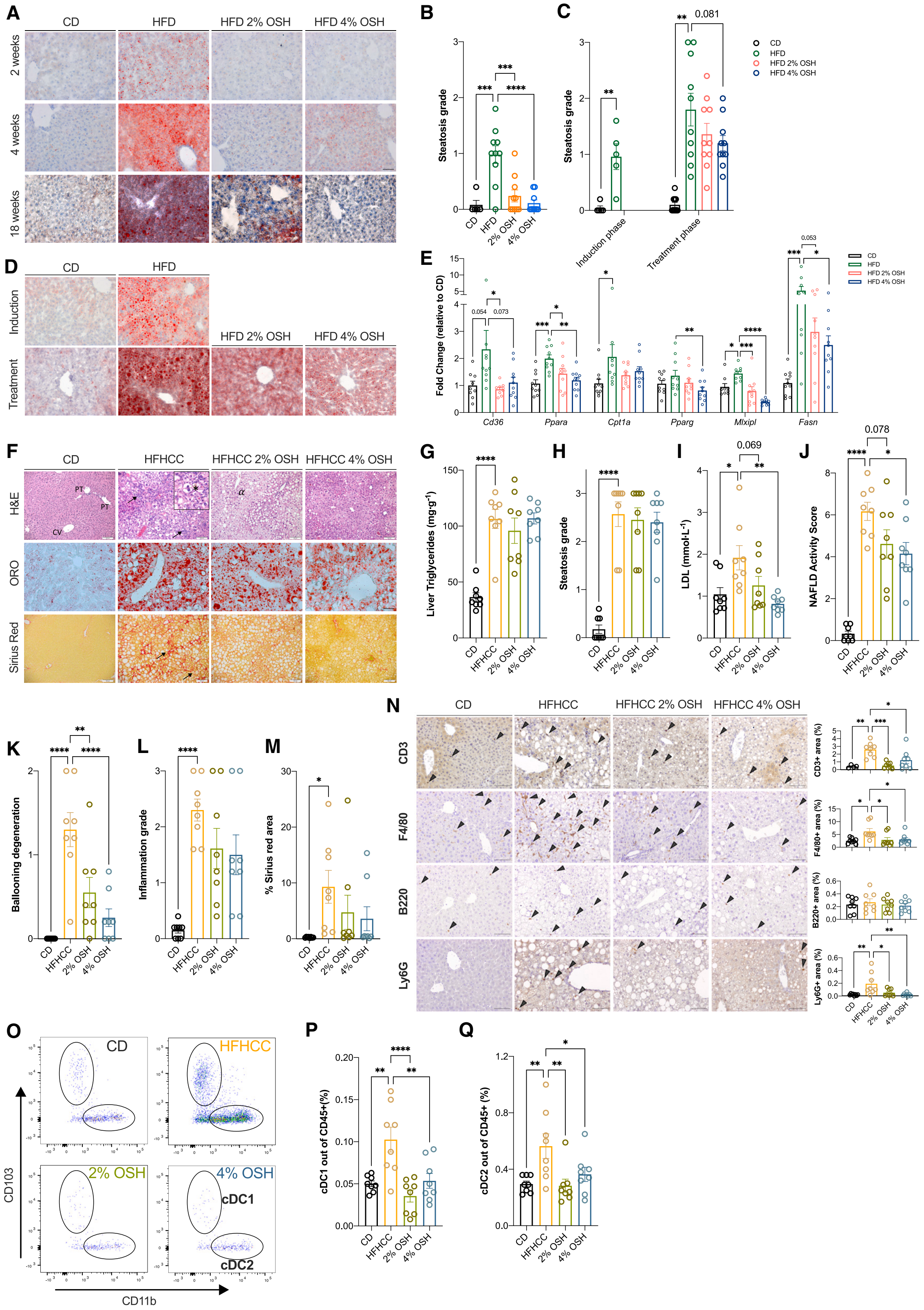

(legend on next page)

3S). Finally, confirming the protective ability of the hydrogel in strengthening gut barrier properties, we observed that Muc-2 was overexpressed in the colon of treated mice (Figures 3T and 3V). Similarly, ileal ZO-1 expression was induced in mice receiving the hydrogel (Figures 3T and 3V). We reasonably argue that the therapeutic administration of OSH, by targeting the intestine and preventing gut permeability, was able to contrast and counteract the alterations of MetS.

OSH limits the progression of non-alcoholic fatty liver disease

Histological assessment of the liver demonstrated pericentral steatosis after 2–4 weeks of HFD feeding, a pattern that has been described in early stages of NAFLD in humans. Along with increasing time of diet (18 weeks), the fat deposits also expanded to the periportal regions of the liver. Preventative administration of OSH impeded fat accumulation in the liver in the short and long term (Figures 4A and 4B). However, therapeutic treatment on HFD-induced NAFLD was not sufficient to clear the liver of steatosis when mice had established NAFLD already at the start of treatment and an unceasing HFD intake (Figures 4C and 4D). Nevertheless, therapeutic OSH administration was able to control further lipid accumulation in the liver and to reduce cholesterol from circulation, also regulating intrahepatic fatty acid metabolism (Figures 4C and 4D). In this regard, OSH was able to normalize HFD-induced changes in the expression of genes regulating fatty acid oxidation (*Ppara* and *Cpt1a*) and *de novo* lipogenesis (*Pparg*, *Fasn*, and *Mlxipl*), paralleling the hepatocyte uptake of fatty acids reflected by changes in *Cd36* expression (Figure 4E).

After observing that OSH could regulate cholesterol metabolism and prevent liver steatosis while maintaining the integrity of the gut barrier, crucial events in the progression of liver disease, we decided to use a NASH model induced by a high-fat, 1% cholesterol, and carbohydrates (fructose/glucose) diet (HFHCC), which closely reproduces both hepatic and extrahepatic manifestations of the human disease.[22] Animals were induced for 10 weeks and then randomized into receiving OSH (2% and 4%) or not for a further 6 weeks together with the HFHCC diet. After 16 weeks of feeding, HFHCC mice had macrovesicular hepatosteatosis accentuated on zone 3, striking lobular inflammation, and hepatocyte degeneration, also showing pericellular/perivenular "chicken-wire" fibrosis, together with peripheral signs of MetS, which are hallmarks of early NASH in humans. As in the HFD experiment, therapeutic administration of OSH after the induction phase was associated with reduced weight gain and metabolic improvement (Figures S3A–S3D). The treatment was unable to abolish liver fat content (Figures 4F–4H); nevertheless, even in this model with extra cholesterol supplementation, we observed a reduction in hypercholesterolemia mainly through lowering of serum LDL (Figures 4I and S3E). We observed an improvement in the NAFLD activity score ($p = 0.078$ for 2%, $p = 0.017$ for 4%), reflecting lower inflammation grade and less hepatocyte damage in the form of ballooning degeneration ($p = 0.003$ for 2%, $p < 0.001$ for 4%) (Figures 4J–4L). According to the IASL system, liver fibrosis was moderate (with occasional septae) in 62.5% (5/8) of mice in the HFHCC group, while only 25% (2/8) of mice in both 2% and 4% groups exhibited liver fibrosis (Pearson's χ^2 $p < 0.05$), reflected also by the Sirius red-stained area, which, however, did not reach statistical significance (Figure 4M).

A deeper analysis into the hepatic inflammatory pattern through immunohistochemistry (IHC) revealed that the treatment was accompanied by a lower accumulation of T cells ($CD3^+$), macrophages ($F4/80^+$), and neutrophils ($Ly6G^+$), with no changes in B cells ($B220^+$) (Figure 4N).

Recent pieces of evidence have placed hepatic dendritic cells as a nexus between lipid metabolism and liver inflammation, playing a crucial role in the progression of metabolic steatohepatitis.[23,24] Flow cytometry analysis of liver myeloid cells revealed that the expansion of conventional type 1 dendritic cells (cDC1) and type 2 (cDC2) induced by HFHCC feeding was prevented with the treatment (Figures 4O–4Q; gating strategy is shown in Figure S3H). This brings to light, in agreement with our previous work, how the protection against early events disrupting the gut barrier affects liver inflammation. In addition to

Figure 4. OSH administration prevents HFD-induced liver steatosis and limits NAFLD progression

(A) Liver tissue sections of C57BL/6J male mice fed for 2 (top), 4 (middle), and 18 (bottom) weeks with an HFD diet or an HFD supplemented with 2% and 4% OSH or CD, stained with oil red O (ORO); representative images of 1 of 5 (2 and 4 weeks) and 1 of 5 (CD) or 9 or 10 mice per group (18 weeks); scale bar, 50 μm.

(B) Steatosis grade histologically quantified after 18 weeks of preventative OSH treatment.

(C) Steatosis grade prior to and after 12 weeks of OSH therapeutic administration.

(D) Liver tissue sections stained with ORO. Top row illustrates one representative image each of liver sections from CD- and HFD-fed mice at the end of disease induction phase (12 weeks HFD, n = 5); bottom row shows liver section images after 12 weeks of OSH therapeutic treatment. Scale bar, 50 μm.

(E) Relative gene expression levels of lipid and fatty acid transporter *Cd36* and regulators of lipid metabolism and β oxidation *Ppara*, *Cpt1a*, *Pparg*, *Mlxipl*, and *Fasn* in the liver after 12 weeks of OSH treatment, expressed as fold change of CD (n = 10).

(F) Liver tissue sections of C57BL/6J male mice therapeutically fed for 6 weeks with OSH-supplemented HFHCC diet, stained with H&E (arrows point to inflammatory foci, the star indicates an example of hepatocyte degeneration, and α indicates microvesicular steatosis surrounding zone 3), ORO for liver triglycerides, and Sirius red for liver fibrosis (arrows point to pericellular and perivenular collagen accumulation). Scale bars, 50 and 100 μm (ORO-H&E and Sirius red, respectively).

(G–I) Liver triglyceride quantification expressed in milligrams per gram of liver tissue, histological quantification of steatosis grade, and circulating LDL cholesterol levels, respectively.

(J–M) NAFLD activity score, ballooning degeneration score, inflammation grade, and percentage of Sirius red-positive area, respectively.

(N) IHC staining on liver tissue sections for CD3, F4/80, Ly6G, and B220. Scale bar, 100 μm, arrowheads indicate DAB-positive cells; relative quantification on the right side.

(O–Q) Fluorescence-activated cell sorting (FACS) analysis of liver immune populations. (O) Gating strategy for cDC1 ($CD11c^+$, $MHCII^+$, and $CD103^+$) and cDC2 ($CD11c^+$, $CD11b^+$, $CD3^-$, and $B220^-$), (P) frequency of cDC1 in $CD45^+$ cells, and (Q) frequency of cDC2 in $CD45^+$ cells (n = 8 mice per group) (*$p < 0.05$; **$p < 0.01$; ***$p < 0.001$; ****$p < 0.0001$, one-way ANOVA Dunnett's post-test, line at mean with SEM; n = 10 mice per group in all graphs if not specified differently).

CellPress
OPEN ACCESS

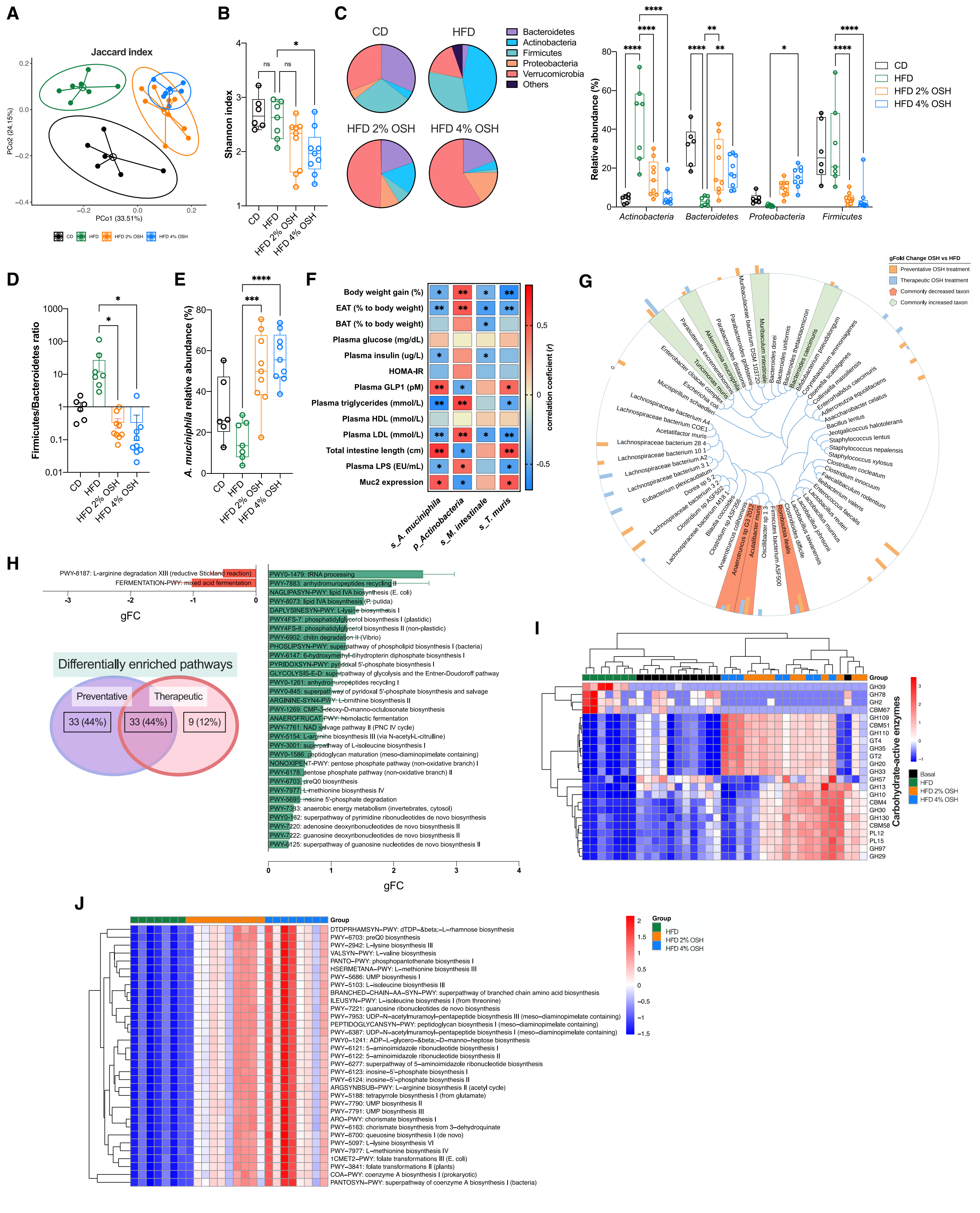

(legend on next page)

normalizing lipid metabolism, OSH was able to control liver inflammation by enhancing gut health, thus limiting the progression of NASH and fibrosis.

To rule out that the observed amelioration of liver steatosis was just secondary to weight loss and systemic changes in metabolism, we used a methionine-choline-deficient diet (MCDD) model. This diet causes a deficit in the production and exportation of VLDL particles, resulting in NASH-like liver histology but lacking systemic metabolic features of the disease. Preventative administration of OSH together with the diet for 4 weeks resulted in reduced liver fat accumulation, decreased serum cholesterol, and prevention of intestinal shortening independent of body weight changes (Figures S4A–S4H).

Hydrogel administration modifies gut microbiota composition and partially prevents HFD-induced dysbiosis

Several studies have placed the gut microbiota at the center of metabolic disorders by controlling different mechanisms, such as energy harvesting and metabolite production, featuring a systemic bioactive effect with both metabolic and inflammatory functions.[9] Since OSH is not systemically absorbed but can be active throughout the intestine, we hypothesized that it could interact with the gut microbiota, eventually changing its composition, abundance, and/or function, and thus we performed shotgun DNA sequencing to address fecal microbiota changes. Principal-coordinate analysis (PCoA) of β diversity revealed a shift in gut microbiota composition in mice undergoing hydrogel administration (Figure 5A). Sequential sampling throughout the experiment showed that these changes happened early and were consistent over time (Figure S5A). α diversity analysis showed a trend toward a lower Shannon index, indicating the expansion and a certain degree of dominance of some species in terms of abundance (Figure 5B). At the phylum level, HFD was associated with a loss of Bacteroidetes (31.5% vs. 3.0%) and a 10-fold expansion of Actinobacteria (4.1% vs. 44.1%), a phylum previously associated with obesity.[25] OSH administration impeded the overgrowth of Actinobacteria and the decrease in Bacteroidetes, mainly at the expense of Firmicutes (30.8% HFD vs. 4.3% HFD 2% OSH and 4.1% HFD 4% OSH; $p < 0.001$) (Figure 5C). Consequently, the ratio of Firmicutes to Bacteroidetes (F/B), a parameter that has been extensively associated with HFD feeding,[8] was restored with the treatment (Figure 5D).

At the species level, we observed a remarkable enrichment in *Turicimonas muris* and *Muribaculum intestinale* abundance in mice with OSH (Figures S5B and S5C). These bacteria were recently described as part of the mouse intestinal bacterial collection.[26,27] Not many shreds of evidence for their role in health and disease have been published since then; the first was found to increase in mice under exercise vs. sedentary conditions,[28] and the latter was shown to be a major forager of mucus-derived monosaccharides.[29] Contrariwise, we observed a rise of *Bifidobacterium pseudolongum* in the HFD group, but not in those with OSH (Figure S5D). However, none of these species are normally present among adult human gut microbiota; so, the more striking result, showing also a stronger correlation with the different clinical parameters, was the increase in *A. muciniphila* abundance in treated animals (Figure 5E). Correlations of the different taxa with relevant disease markers are shown in Figure 5F. Similar results were recapitulated in mice that therapeutically received OSH (Figures 5G and S5E–S5J). Changes were also noticeable in the ileum microbiota (Figure S5K).

Whole metagenome annotation revealed that the changes in composition were associated with a potential shift in functionality, with up to 75 differentially enriched pathways upon preventative treatment, 33 of which were recapitulated in the therapeutic setting (Figure 5H). Interestingly, we found an overall increase in the carbohydrate-processing capacity reflected by the abundance of 19 families of carbohydrate-active enzymes (CAZymes) (Figure 5I). Among them, in the OSH groups, there was an increase in mucin-associated glycoside hydrolases and glycosyltransferases (GH20, GH29, GH30, GH33, GH35, GH109, GT2, and GT4) and carbohydrate-binding modules (CBM4, CBM51, and CBM58) with the capacity of processing starch, cellulose and other plant cell-wall-derived polysaccharides, expanding the metabolic potential that enables gut bacteria to utilize dietary fibers and mucosal glycans. Finally, we observed an enrichment in *Akkermansia* pathways in treated animals compared with HFD alone, which correlates and further confirms the increased levels of this bacterium and its metabolic activity (Figure 5J).

Overall, these data show that hydrogel administration can modulate the gut microbiota, preventing those microbiota

Figure 5. OSH administration shapes gut microbiota composition, preventing and reversing HFD-induced alterations

(A) PCoA of fecal microbiota from mice fed for 18 weeks HFD (green) or OSH-supplemented HFD (2% OSH, orange, and 4% OSH, light blue), compared with basal (CD, black), based on the Jaccard index; single dots represent individual mice (PERMANOVA, $p < 0.001$).

(B) Shannon's diversity index of fecal microbiota from mice fed for 18 weeks with OSH-supplemented HFD (*$p < 0.05$; one-way ANOVA, Dunnett's *post hoc* test).

(C) Relative abundance of detected fecal bacteria phyla (*$p < 0.05$; **$p < 0.01$; ***$p < 0.001$; ****$p < 0.0001$; one-way ANOVA, Dunnett's *post hoc* test).

(D) Firmicutes/Bacteroidetes ratio.

(E) *Akkermansia muciniphila* relative abundance.

(F) Correlation between bacterial taxa with metabolic variables measured after 18 weeks of preventative OSH feeding (*$p < 0.05$; **$p < 0.01$; Spearman correlation).

(G) Cladogram including all bacterial species identified in fecal metagenome. Colored bar plot shows differences between OSH supplemented groups and HFD expressed as log fold change of bacterial abundance (preventative study in orange and therapeutic study in light blue; commonly decreased taxon in red and commonly increased taxon in green).

(H) Functional analysis. Venn diagram and plots of differentially abundant pathways showing differences between OSH-supplemented groups and HFD commonly found in preventative and therapeutic mouse studies with a gFC > |0.3|; the bar plot represents the change in pathway abundance (gFC) and the error bars refer to the variability between the preventative and the therapeutic experiments.

(I) Heatmap of differentially abundant carbohydrate-active enzymes (CAZymes).

(J) Heatmap of *Akkermansia muciniphila*-associated pathways.

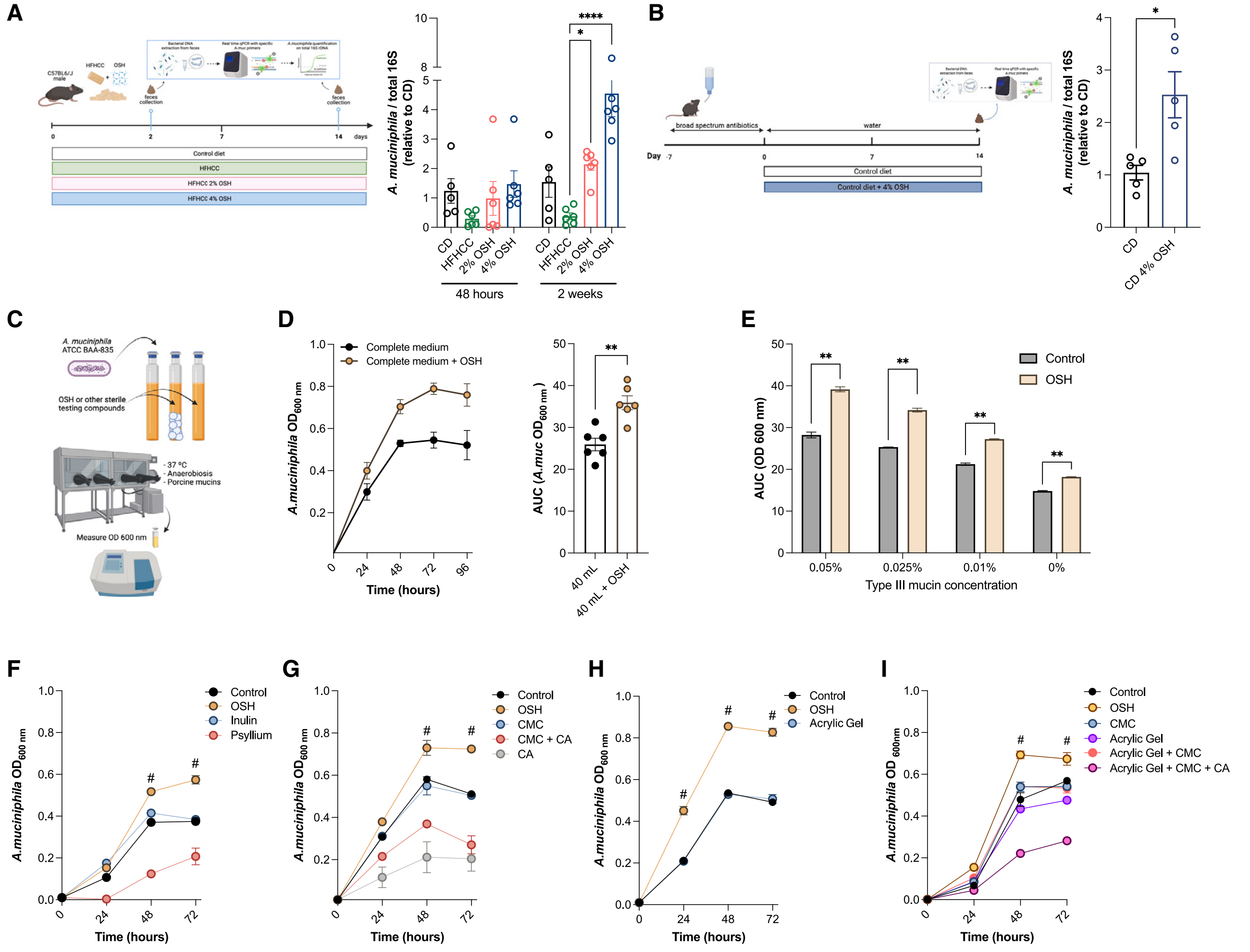

Figure 6. OSH abets rapid *Akkermansia muciniphila* expansion both *in vivo* and *in vitro*

(A) Experimental scheme for short-term *A. muciniphila* abundance quantification upon HFHCC and OSH administration. Relative *A. muciniphila* abundance was normalized to the total 16S rRNA levels (n = 5 mice/group).

(B) Fecal *A. muciniphila* relative abundance (normalized to total 16S rRNA) 2 weeks after microbiota depletion with a broad-spectrum antibiotic cocktail.

(C) Scheme for *in vitro* culture of *A. muciniphila*.

(D) *A. muciniphila* growth curve expressed as optical density at 600 nm wavelength (OD_{600nm}) in the presence or absence of OSH; AUC (n = 6 repetitions), unpaired t test, $^{**}p < 0.01$.

(E) AUC of *A. muciniphila* growth curves with decreasing concentrations of type III porcine mucin (0.05%–0%). Multiple unpaired t test, $^{**}p < 0.01$; line at mean with SEM.

(F) *A. muciniphila* growth in the presence of OSH vs. fermentable fiber inulin and gel-forming fiber psyllium.

(G) *A. muciniphila* growth curve in presence of OSH vs. OSH individual components: carboxymethyl cellulose (CMC), citric acid (CA), and the two components together un-cross-linked.

(H) *A. muciniphila* growth curve in presence of OSH vs. a gel-forming compound of synthetic origin.

(I) *A. muciniphila* growth curve expressed as OD_{600nm} in the presence of OSH vs. a gel-forming compound of synthetic origin and combination of the last with OSH individual components (CMC and CA and CMC + CA). (F–I) $^{\#}p < 0.0001$, one- or two-way ANOVA, Dunnett's post-test relative to OSH vs. control, inulin, and psyllium, mean of two independent tests, line at mean with SEM.

alterations and dysbiosis typical of HFD and "westernized" dietary habits.

OSH fosters *A. muciniphila* growth via its physical and chemical properties

One of the major and consistent changes in the gut microbiota composition was the increase in the abundance of *A. muciniphila*, a bacterium that has been shown to protect from metabolic disorders by reducing insulin resistance, fat-mass gain, inflammation, and endotoxemia.[13,30] To test whether this was a secondary or direct effect of OSH, we analyzed the impact on *Akkermansia* abundance by a short-term OSH intervention in HFHCC-fed mice. The HFHCC diet rapidly (48 h) decreased *Akkermansia* abundance compared with chow-fed animals, while supplementation with OSH prevented this reduction (Figure 6B). After 2 weeks, *A. muciniphila* was 5.7- and 12.0-fold more

abundant in animals supplemented with 2% and 4% OSH, respectively, than in those on the HFHCC diet alone (Figure 6A). In a different experiment, to assess the role of the existing microbiota in supporting *Akkermansia* growth in response to OSH treatment, we first depleted the gut microbiota of mice fed a CD by using broad-spectrum antibiotics for 1 week. Even starting with a disrupted microbiota, 2 weeks of OSH treatment allowed for an enrichment of *Akkermansia* (2.52 times compared with CD alone; p = 0.012) (Figure 6B).

Altogether, these results suggest that OSH administration directly sustains *A. muciniphila* growth in the presence or absence of a complete microbiota.

To corroborate a direct action of OSH in fostering *Akkermansia* growth, we designed an *in vitro* anaerobic system to study bacterial (ATCC-BAA-835) growth in the presence or absence of OSH by measuring the OD 600 nm of the culture in time. Our results showed that *A. muciniphila* growth was progressively stimulated by the presence of OSH in culture (Figures 6C and 6D). This bacterium is a mucus degrader that resides in the mucus layer of the large intestine, and its culture is highly mucin dependent.[31] We thus cultured the bacterium in medium with decreasing concentrations of mucins (0.05%, 0.025%, 0.01%, and 0%) and observed a beneficial effect of OSH in bacterial growth even in the total absence of mucins (Figure 6E).

In addition, we tested if the growth could be stimulated by other compounds, such as inulin, an herbal polysaccharide largely used as a prebiotic, or psyllium, a gel-forming soluble fiber made from the husks of *Plantago* spp. plant seeds, both of which have been reported to reduce serum cholesterol levels in hyperlipidemic subjects and improve glycemic control in type 2 diabetes.[32–34] Our results demonstrated that inulin addition *in vitro* gives no advantage to *Akkermansia* growth over the control medium, remaining significantly lower than the effect of OSH (p < 0.001 after 48 h), while psyllium fiber seemed to even reduce the growth of *Akkermansia* (p < 0.001vs. OSH after 48 h) (Figure 6F).

Finally, to explore whether this effect was mediated by the chemical properties of OSH or by its cross-linked three-dimensional (3D) structure, we tested its components individually (carboxymethyl cellulose [CMC] and citric acid [CA]) or together, in an un-cross-linked formulation. CMC alone showed no difference from the control, while CA alone, which is used as the cross-linker, inhibited the growth of the bacterium with or without free CMC (Figure 6G). Further, it was not just the physical properties that conferred the *Akkermansia* growth-promoting ability to OSH, as we observed that another gel-forming compound with similar 3D structure and elasticity, but of synthetic origin, showed no benefit for *A. muciniphila* growth compared with the control (Figure 6H), as neither did the combination of its individual chemical components together with the 3D structure created by the synthetic gel (Figure 6I). Thus, the capacity of OSH to boost *Akkermansia* growth relies on both physical and chemical properties (i.e., a degradable fiber [CMC] in a 3D cellulose-based matrix with high water content and high elasticity levels, which mimics raw vegetables) present in a single entity.[15]

Gut microbiota drives metabolic benefits of OSH in mice

To understand if the changes in gut microbiota were responsible for the metabolic improvement observed in the different animal models, we performed a fecal microbiota transplantation (FMT). To do so, we selected as microbiota donors male C57BL6/J mice treated or not for 2 weeks with 2% or 4% OSH (time at which we observed a blooming of *A. muciniphila*; Figure 6A) after 10 weeks of feeding with HFHCC diet to induce MetS and NAFLD with liver inflammation. The fecal material was freshly collected, and 20 mg/mouse was transferred via gavage every other day for a total of 4 weeks into recipient animals. The latter were age-paired mice induced with HFHCC diet for 12 weeks and maintained on the same diet throughout the FMT (scheme in Figure 7A).

The 12-week HFHCC diet led to obesity (29.8 g chow vs. 40.4 g HFHCC; p < 0.001) and to glucose intolerance (AUC GTT p = 0.039) (data not shown). After 4 weeks of FMT, recipient mice from OSH-treated animals lost 3.8% of basal weight for 2% OSH and lost 2.5% for 4% OSH, while those receiving FMT from HFHCC control animals continued to gain weight (7.0% from baseline) (Figure 7B). In addition, the GTT and ITT were significantly improved after allogeneic microbiota transplantation (Figures 7C and 7D).

The FMT from OSH-treated donors was also associated with better liver outcomes, as they exhibited reduced liver mass enlargement (hepatomegaly) and significantly decreased plasma ALT levels (Figures 7E and 7F). Histologically, and in line with this, they displayed lower hepatosteatosis, accompanied by a difference in the NAFLD activity score of −1.47 and −1.65 points (2% and 4%, respectively), reflecting altogether an improvement in HFHCC-induced liver damage (Figures 7G–7I). Regarding circulating cholesterol levels, we observed the expected trend toward a reduction in LDL, although not reaching statistical significance, probably due to the limited sample size (Figure 7J).

Nevertheless, in contrast with mice receiving the treatment (Figures S1F, S3F, S3G, S4D, 2, and 3R–3V), we did not find recovery in the shortening of the intestine, and the functional analysis of the gut barrier disruption showed no significant improvement in either the FITC-dextran assay or the LPS levels after allogeneic FMT (Figures S6A–S6C). Altogether, these data indicate an effect of the gel in promoting the development of a MetS-protecting microbiota. However, they also highlight a direct effect of the OSH on the intestinal barrier, which is not observable with just FMT, and suggest a dynamic exoskeleton protective role of the gel based on its mechanochemical properties, in agreement with our recent publication.[15] Therefore, the physical properties exerted by the presence of the OSH in the GI lumen, together with the microbiota changes, act synergistically and are necessary to observe the full spectrum of metabolic benefits conferred by the OSH treatment in mice.

DISCUSSION

In this study, we describe the beneficial metabolic effects engendered by the use of a biomimetic superabsorbent hydrogel as an oral, non-systemic approach for the treatment of MetS and its associated disorders.

The utilization of superabsorbent hydrogel technologies as a non-systemic therapy to enhance satiety and assist in weight management is a growing area of interest. Different approaches, currently at various stages of preclinical and clinical

CellPress
OPEN ACCESS

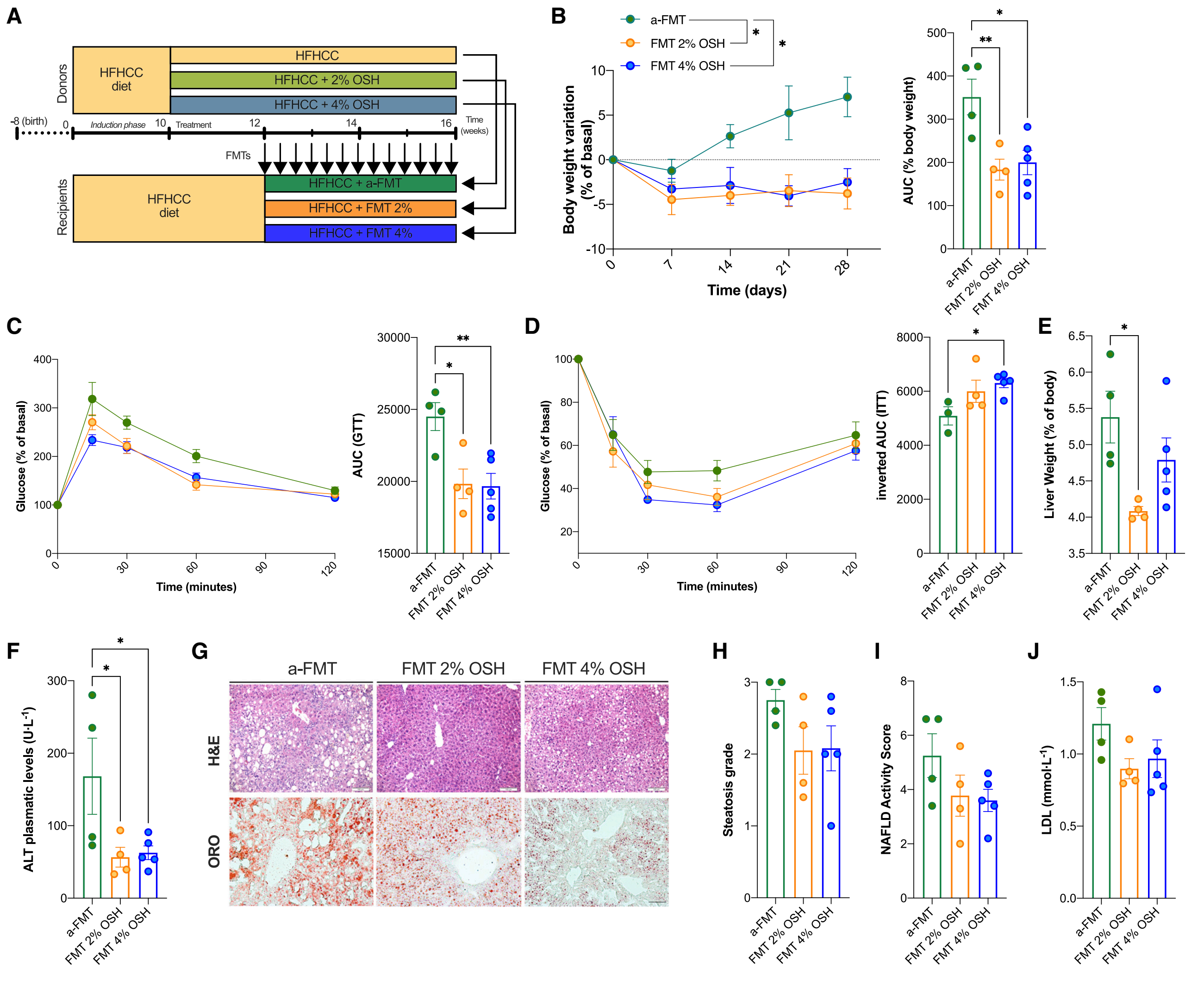

Figure 7. FMT of OSH-shaped gut microbiota reduces body weight and restores glucose and insulin metabolism in HFHCC-fed mice

(A) Experimental design of the FMT.

(B) Recipients' body weight variation expressed as percentage of basal, where basal is the weight before the first FMT oral gavage; AUC of body-weight variation curve.

(C and D) Intraperitoneal GTT and ITT, respectively.

(E) Liver weight.

(F) Alanine aminotransferase (ALT) serum levels in recipient mice after 4 weeks of FMT.

(G) Liver histology. H&E and ORO staining for liver triglycerides. Scale bars, 50 and 100 μm (ORO and H&E).

(H and I) Histological score of steatosis grade and NAFLD activity score, respectively, after 4 weeks of FMT.

(J) Recipients' serum LDL levels (*$p < 0.05$ and **$p < 0.01$, one-way ANOVA, Dunnett's post-test, line at mean with SEM; number of mice per group n = 4 autologous FMT [HFHCC in HFHCC-fed recipients], n = 4 FMT 2% OSH in HFHCC recipients, n = 5 FMT 4% OSH in HFHCC recipients in A–J).

development, have been described to date.[35,36] Key characteristics make OSH unique compared with other known gastric space-filling devices of synthetic origin, including also gastric balloons, which partially and temporarily fill the stomach volume to induce satiety. By contrast, OSH is designed to form individual particles that, upon hydration, homogeneously mix with the food, mimicking the composition and mechanical properties of fiber-rich raw vegetables without adding calories and, as demonstrated in this work, also have protective effects throughout the entire digestive system.

To the best of our knowledge, this is the only superabsorbent hydrogel produced using naturally derived building blocks that are also used as food ingredients. By using CMC as the backbone of a cross-linked hydrogel, a highly favorable safety and tolerability profile was demonstrated, accompanied by a beneficial metabolic response in both rodents and humans.[37] This is likely due to the fact that, with the 3D structure of a hydrogel, its CMC component is not functioning anymore as an emulsifying agent. Data from the latest clinical trial (NCT03058029) confirmed a similar incidence of treatment-emergent adverse

events compared with placebo, without changes in macro- or micronutrients, blood laboratory tests, or serum vitamins, while being effective in lowering body weight in people who are overweight or obese.[37]

In addition to weight control, we show here that OSH was able to regulate glucose and lipid metabolism, improving insulin resistance and controlling adipose tissue hypertrophy, circulating cholesterol levels, and liver steatosis accompanied by lower liver inflammatory infiltration, ultimately ameliorating MetS and limiting the progression of NAFLD. In light of our results, we postulate that two non-mutually exclusive mechanisms could underlie the metabolic benefits occurring upon hydrogel administration. First, the hydrogel could increase the barrier properties of intestinal tissues, like a dynamic exoskeleton, protecting from HFD-induced increase in gut barrier permeability and preventing systemic endotoxemia, thus indirectly limiting inflammatory processes that would exacerbate MetS progression. Second, it induces consistent and unique gut microbiota changes, preventing or improving HFD-induced dysbiosis, with a specific sway on *A. muciniphila* abundance, which has been inversely correlated to obesity, type 2 diabetes, or NAFLD, among other pathological conditions.[38] We recently described that the gut-protecting properties of OSH rely on its elasticity, obtained after chemical bonding of the matrix (CMC) to form a 3D network by means of strong primary bonds, preventing flow as a viscous liquid with a consistency typical of raw vegetables.[15] These elastic characteristics and 3D structure distinguish OSH from a typical prebiotic fiber, which may exhibit a viscous flow once mixed with water solutions and display negligible elastic modulus. CMC, for instance, is known to have a limited level of fermentation in the gut.[39] Indeed, we observed that non-cross-linked CMC does not by itself promote *A. muciniphila* growth, nor does inulin or psyllium. Therefore, both the mechanical and the chemical properties created by the 3D structure of OSH are required to allow *Akkermansia* growth, as a non-degradable acrylic gel, with a similar elasticity, was unable to promote *Akkermansia* growth.

Indeed, the cross-linked CMC creates a superabsorbent hydrogel with distinct elastic properties in the hydrated state, as opposed to those of un-cross-linked CMC or other dietary fiber supplements (e.g., glucomannan and psyllium) that rather form viscous liquids when hydrated. OSH exhibits an elasticity that is orders of magnitude higher than those of dietary fiber supplements or other OSH products and is more similar to that of natural, fiber-rich vegetables.[15] These physicochemical properties could affect the GI tract in regard to weight regulation, such as gastric distention and emptying or GI transit time, which may promote mechanical- and hormone-mediated satiety.[40–42] Recent literature supports that the capacity of the gut to sense fatty acids (FAs) is compromised in obese patients, with an expression of *CD36*, the receptor of FA uptake, around 240-fold higher than that of lean individuals.[43] In this study, we observed a reduction in *Cd36* levels in mice treated with OSH compared with mice fed HFD alone, which could suggest a decrease in FA absorption. It has also been demonstrated that increased FA sensing could have an impact on the release of GI hormones affecting the exocrine pancreas and insulin secretion,[44] which is in agreement with the reduction in fatty acid transporters and insulinemia we observed after treatment with the hydrogel. By contrast, higher insulin levels could enhance liver *Cd36* expression and induce steatosis and hepatic insulin resistance.[45] Although we found no significant differences in gut transit time, energy absorption, or food intake, we cannot completely exclude that these mechanisms play a role in the observed effects; however, the finding that FMT was able to recapitulate the MetS-protecting effects of the gel might argue against it.

The gut microbiota has been shown to play a major role in obesity-related disorders by controlling the energy balance and influencing whole-body metabolism.[12] A recent meta-analysis showed reproducible alterations in gut microbiota due to HFD, among which the F/B ratio was found to be increased across studies.[46] Administration of the hydrogel restored the F/B ratio and induced *A. muciniphila* expansion, which correlated with weight loss without observing changes in the total energy ingested. *A. muciniphila*, even in a pasteurized form, can counteract diet-induced obesity through the modulation of energy expenditure in mice and improve glucose homeostasis and metabolic disease by directly inducing GLP-1 secretion.[47,48] GLP-1 receptor agonism has been associated with a reduction in body weight and the resolution of NASH.[49–51] Consistently, we observed an increase in GLP-1 coupled to *Akkermansia* expansion, which could be a major driver of weight loss by increasing whole-body energy expenditure.[47]

Targeting the microbiota may represent new avenues for therapeutic interventions aimed at preventing or treating metabolic disorders. Nevertheless, although the use of live biotherapeutic products (either as probiotics or as FMT) is an area of exponential growth, several factors, including risk-to-benefit ratio, are yet limiting their approval and wide application[52,53]; therefore, while waiting to overcome these barriers, hydrogel administration could be used as a non-invasive strategy to both increase *A. muciniphila* abundance *in vivo* and ameliorate HFD-induced gut dysbiosis.

We conclude that OSH promotes gut microbiota changes, inducing the expansion of species that have been reported to be beneficial for intact metabolic function (i.e., *A. muciniphila*). According to our *in vitro* results, not only its chemical composition but also its physical structure provide OSH with the unique capability to boost *Akkermansia* growth, even in the absence of mucins. We also confirm a direct interplay between OSH and the gut, playing a role in intestinal tissue preservation and mucosal homeostasis by affecting both mucosal and epithelial barriers. So, the biomimetic mechanical properties of OSH counteracted the translocation of bacteria and their subproducts and the subsequent low-grade inflammation promoted by HFD feeding, which makes a well-established contribution to the pathophysiological processes underlying obesity, insulin resistance onset, and NASH.[7,54,55] Our work shows the potential of using OSH in the treatment of obesity and metabolic disorders as an effective, natural, and non-invasive therapeutic tool. However, clinical studies will be needed to replicate these findings in humans and to evaluate patient adherence together with long-term success of OSH treatment.

Limitations of the study

This study faces limitations in the extrapolation of the obtained results from mice to humans, where assumptions on metabolism,

energy harvest, and feeding pattern similarity are made, while there are intrinsic physiological, anatomical, and genetic differences across species. In accordance, dissimilarities in the composition of the gut microbiota cannot exclude that other species beyond *A. muciniphila* can be affected by OSH treatment.

STAR★METHODS

Detailed methods are provided in the online version of this paper and include the following:

- KEY RESOURCES TABLE
- RESOURCE AVAILABILITY
  - Lead contact
  - Materials availability
  - Data and code availability
- EXPERIMENTAL MODEL AND STUDY PARTICIPANT DETAILS
  - Animals
  - Microbe strain
- METHOD DETAILS
  - OSH synthesis and diet preparation
  - Histopathological assessment
  - FITC-dextran permeability assay
  - Glucose tolerance and Insulin Tolerance Tests
  - Carmine red dye gut transit time assay
  - Biochemical analysis in serum/plasma
  - Hepatic triglycerides quantification
  - Quantification of DNA and mRNA by real-time PCR
  - Flow cytometry
  - Fecal microbiota transplantation
  - Gut metagenomic analysis
- QUANTIFICATION AND STATISTICAL ANALYSIS

SUPPLEMENTAL INFORMATION

Supplemental information can be found online at https://doi.org/10.1016/j.xcrm.2023.101235.

ACKNOWLEDGMENTS

The research leading to these results has received funding from Gelesis S.r.l.; Gelesis is a biotechnology company that develops novel therapies for gastrointestinal-related chronic diseases. We acknowledge a fellowship from the Consejería de Salud y Familias, Junta de Andalucía, to A.G.-G. (RH-122-2020). The graphical abstract was created using BioRender. We would like to thank Marta Salvi for technical support.

AUTHOR CONTRIBUTIONS

Conceptualization, A. Silvestri, A.G.-G., M.V., C.D., B.J., J.M., G.P., A. Sannino, and M.R.; methodology, investigation, formal analysis, and data curation, A. Silvestri, A.G.-G., and M.V.; investigation, P.B., I.S., G.F., J.M., S.C., and M.L.; writing, A. Silvestri, A.G.-G., M.M., and M.R.; software and formal analysis, D.B.; resources, C.D., M.M., B.J., and M.R.-G.; supervision, A. Sannino and M.R.; project administration, M.R.; funding acquisition, M.R.

DECLARATION OF INTERESTS

A. Silvestri, A.G.-G., M.V., D.B., P.B., I.S., G.F., J.M., S.C., M.L., M.R.-G., G.P., and M.R. declare that this work has been supported by funding provided by Gelesis S.r.l. B.J. is employed by Gelesis, Inc., and owns stock options. C.D., M.M., and A. Sannino are employed by Gelesis S.r.l.; C.D., A. Sannino, and M.R. own Gelesis, Inc., stock options. M.R., A. Silvestri, A. Sannino, and C.D. are inventors on patent US 10695363 "Compositions and methods for treating or preventing gut permeability-related disorders" (applicant: GELESIS LLC, Boston MA, USA).

INCLUSION AND DIVERSITY

We support inclusive, diverse, and equitable conduct of research.

Received: February 17, 2023
Revised: July 31, 2023
Accepted: September 19, 2023
Published: October 17, 2023

REFERENCES

1. Blüher, M. (2019). Obesity: global epidemiology and pathogenesis. Nat. Rev. Endocrinol. *15*, 288–298. https://doi.org/10.1038/s41574-019-0176-8.
2. Camilleri, M., and Vella, A. (2022). What to do about the leaky gut. Gut *71*, 424–435. https://doi.org/10.1136/gutjnl-2021-325428.
3. GBD 2015 Obesity Collaborators; Afshin, A., Forouzanfar, M.H., Reitsma, M.B., Sur, P., Estep, K., Lee, A., Marczak, L., Mokdad, A.H., Moradi-Lakeh, M., et al. (2017). Health Effects of Overweight and Obesity in 195 Countries over 25 Years. N. Engl. J. Med. *377*, 13–27. https://doi.org/10.1056/NEJMoa1614362.
4. Ward, Z.J., Bleich, S.N., Cradock, A.L., Barrett, J.L., Giles, C.M., Flax, C., Long, M.W., and Gortmaker, S.L. (2019). Projected U.S. State-Level Prevalence of Adult Obesity and Severe Obesity. N. Engl. J. Med. *381*, 2440–2450. https://doi.org/10.1056/NEJMsa1909301.
5. Bhaskaran, K., Dos-Santos-Silva, I., Leon, D.A., Douglas, I.J., and Smeeth, L. (2018). Association of BMI with overall and cause-specific mortality: a population-based cohort study of 3·6 million adults in the UK. Lancet Diabetes Endocrinol. *6*, 944–953. https://doi.org/10.1016/s2213-8587(18)30288-2.
6. Müller, T.D., Blüher, M., Tschöp, M.H., and DiMarchi, R.D. (2022). Anti-obesity drug discovery: advances and challenges. Nat. Rev. Drug Discov. *21*, 201–223. https://doi.org/10.1038/s41573-021-00337-8.
7. Mouries, J., Brescia, P., Silvestri, A., Spadoni, I., Sorribas, M., Wiest, R., Mileti, E., Galbiati, M., Invernizzi, P., Adorini, L., et al. (2019). Microbiota-driven gut vascular barrier disruption is a prerequisite for non-alcoholic steatohepatitis development. J. Hepatol. *71*, 1216–1228. https://doi.org/10.1016/j.jhep.2019.08.005.
8. Ley, R.E., Turnbaugh, P.J., Klein, S., and Gordon, J.I. (2006). Human gut microbes associated with obesity. Nature *444*, 1022–1023. https://doi.org/10.1038/4441022a.
9. Fan, Y., and Pedersen, O. (2021). Gut microbiota in human metabolic health and disease. Nat. Rev. Microbiol. *19*, 55–71. https://doi.org/10.1038/s41579-020-0433-9.
10. Aron-Wisnewsky, J., Vigliotti, C., Witjes, J., Le, P., Holleboom, A.G., Verheij, J., Nieuwdorp, M., and Clément, K. (2020). Gut microbiota and human NAFLD: disentangling microbial signatures from metabolic disorders. Nat. Rev. Gastroenterol. Hepatol. *17*, 279–297. https://doi.org/10.1038/s41575-020-0269-9.
11. Cani, P.D., Amar, J., Iglesias, M.A., Poggi, M., Knauf, C., Bastelica, D., Neyrinck, A.M., Fava, F., Tuohy, K.M., Chabo, C., et al. (2007). Metabolic endotoxemia initiates obesity and insulin resistance. Diabetes *56*, 1761–1772. https://doi.org/10.2337/db06-1491.
12. Turnbaugh, P.J., Ley, R.E., Mahowald, M.A., Magrini, V., Mardis, E.R., and Gordon, J.I. (2006). An obesity-associated gut microbiome with increased capacity for energy harvest. Nature *444*, 1027–1031.
13. Depommier, C., Everard, A., Druart, C., Plovier, H., Van Hul, M., Vieira-Silva, S., Falony, G., Raes, J., Maiter, D., Delzenne, N.M., et al. (2019).

Supplementation with Akkermansia muciniphila in overweight and obese human volunteers: a proof-of-concept exploratory study. Nat. Med. *25*, 1096–1103.

14. Greenway, F.L., Aronne, L.J., Raben, A., Astrup, A., Apovian, C.M., Hill, J.O., Kaplan, L.M., Fujioka, K., Matejkova, E., Svacina, S., et al. (2019). A Randomized, Double-Blind, Placebo-Controlled Study of Gelesis100: A Novel Nonsystemic Oral Hydrogel for Weight Loss. Obesity *27*, 205–216. https://doi.org/10.1002/oby.22347.
15. Madaghiele, M., Demitri, C., Surano, I., Silvestri, A., Vitale, M., Panteca, E., Zohar, Y., Rescigno, M., and Sannino, A. (2021). Biomimetic cellulose-based superabsorbent hydrogels for treating obesity. Sci. Rep. *11*, 21394. https://doi.org/10.1038/s41598-021-00884-5.
16. Reister, E.J., and Mattes, R.D. (2022). No apparent effects of a viscous, superabsorbent hydrogel on appetite, energy intake, or fecal excretion in overweight adults. Physiol. Behav. *243*, 113643. https://doi.org/10.1016/j.physbeh.2021.113643.
17. Beyaz, S., Mana, M.D., Roper, J., Kedrin, D., Saadatpour, A., Hong, S.-J., Bauer-Rowe, K.E., Xifaras, M.E., Akkad, A., Arias, E., et al. (2016). High-fat diet enhances stemness and tumorigenicity of intestinal progenitors. Nature *531*, 53–58. https://doi.org/10.1038/nature17173.
18. Garidou, L., Pomié, C., Klopp, P., Waget, A., Charpentier, J., Aloulou, M., Giry, A., Serino, M., Stenman, L., Lahtinen, S., et al. (2015). The Gut Microbiota Regulates Intestinal CD4 T Cells Expressing RORγt and Controls Metabolic Disease. Cell Metabol. *22*, 100–112. https://doi.org/10.1016/j.cmet.2015.06.001.
19. Paone, P., and Cani, P.D. (2020). Mucus barrier, mucins and gut microbiota: the expected slimy partners? Gut *69*, 2232–2243. https://doi.org/10.1136/gutjnl-2020-322260.
20. Harnik, Y., Buchauer, L., Ben-Moshe, S., Averbukh, I., Levin, Y., Savidor, A., Eilam, R., Moor, A.E., and Itzkovitz, S. (2021). Spatial discordances between mRNAs and proteins in the intestinal epithelium. Nat. Metab. *3*, 1680–1693. https://doi.org/10.1038/s42255-021-00504-6.
21. Cani, P.D., Possemiers, S., Van de Wiele, T., Guiot, Y., Everard, A., Rottier, O., Geurts, L., Naslain, D., Neyrinck, A., Lambert, D.M., et al. (2009). Changes in gut microbiota control inflammation in obese mice through a mechanism involving GLP-2-driven improvement of gut permeability. Gut *58*, 1091–1103. https://doi.org/10.1136/gut.2008.165886.
22. Im, Y.R., Hunter, H., de Gracia Hahn, D., Duret, A., Cheah, Q., Dong, J., Fairey, M., Hjalmarsson, C., Li, A., Lim, H.K., et al. (2021). A Systematic Review of Animal Models of NAFLD Finds High-Fat, High-Fructose Diets Most Closely Resemble Human NAFLD. Hepatology *74*, 1884–1901. https://doi.org/10.1002/hep.31897.
23. Haas, J.T., Vonghia, L., Mogilenko, D.A., Verrijken, A., Molendi-Coste, O., Fleury, S., Deprince, A., Nikitin, A., Woitrain, E., Ducrocq-Geoffroy, L., et al. (2019). Transcriptional Network Analysis Implicates Altered Hepatic Immune Function in NASH development and resolution. Nat. Metab. *1*, 604–614. https://doi.org/10.1038/s42255-019-0076-1.
24. Deczkowska, A., David, E., Ramadori, P., Pfister, D., Safran, M., Li, B., Giladi, A., Jaitin, D.A., Barboy, O., Cohen, M., et al. (2021). XCR1+ type 1 conventional dendritic cells drive liver pathology in non-alcoholic steatohepatitis. Nat. Med. *27*, 1043–1054. https://doi.org/10.1038/s41591-021-01344-3.
25. Turnbaugh, P.J., Hamady, M., Yatsunenko, T., Cantarel, B.L., Duncan, A., Ley, R.E., Sogin, M.L., Jones, W.J., Roe, B.A., Affourtit, J.P., et al. (2009). A core gut microbiome in obese and lean twins. Nature *457*, 480–484. https://doi.org/10.1038/nature07540.
26. Lagkouvardos, I., Pukall, R., Abt, B., Foesel, B.U., Meier-Kolthoff, J.P., Kumar, N., Bresciani, A., Martínez, I., Just, S., Ziegler, C., et al. (2016). The Mouse Intestinal Bacterial Collection (miBC) provides host-specific insight into cultured diversity and functional potential of the gut microbiota. Nat. Microbiol. *1*, 16131. https://doi.org/10.1038/nmicrobiol.2016.131.
27. Lagkouvardos, I., Lesker, T.R., Hitch, T.C.A., Gálvez, E.J.C., Smit, N., Neuhaus, K., Wang, J., Baines, J.F., Abt, B., Stecher, B., et al. (2019). Sequence and cultivation study of Muribaculaceae reveals novel species, host preference, and functional potential of this yet undescribed family. Microbiome *7*, 28. https://doi.org/10.1186/s40168-019-0637-2.
28. Dowden, R.A., McGuinness, L.R., Wisniewski, P.J., Campbell, S.C., Guers, J.J., Oydanich, M., Vatner, S.F., Häggblom, M.M., and Kerkhof, L.J. (2020). Host genotype and exercise exhibit species-level selection for members of the gut bacterial communities in the mouse digestive system. Sci. Rep. *10*, 8984. https://doi.org/10.1038/s41598-020-65740-4.
29. Pereira, F.C., Wasmund, K., Cobankovic, I., Jehmlich, N., Herbold, C.W., Lee, K.S., Sziranyi, B., Vesely, C., Decker, T., Stocker, R., et al. (2020). Rational design of a microbial consortium of mucosal sugar utilizers reduces Clostridiodes difficile colonization. Nat. Commun. *11*, 5104. https://doi.org/10.1038/s41467-020-18928-1.
30. Everard, A., Belzer, C., Geurts, L., Ouwerkerk, J.P., Druart, C., Bindels, L.B., Guiot, Y., Derrien, M., Muccioli, G.G., Delzenne, N.M., et al. (2013). Cross-talk between Akkermansia muciniphila and intestinal epithelium controls diet-induced obesity. Proc. Natl. Acad. Sci. USA *110*, 9066–9071.
31. Kim, J.-S., Kang, S.W., Lee, J.H., Park, S.-H., and Lee, J.-S. (2022). The evolution and competitive strategies of Akkermansia muciniphila in gut. Gut Microb. *14*, 2025017. https://doi.org/10.1080/19490976.2021.2025017.
32. Chambers, E.S., Byrne, C.S., Morrison, D.J., Murphy, K.G., Preston, T., Tedford, C., Garcia-Perez, I., Fountana, S., Serrano-Contreras, J.I., Holmes, E., et al. (2019). Dietary supplementation with inulin-propionate ester or inulin improves insulin sensitivity in adults with overweight and obesity with distinct effects on the gut microbiota, plasma metabolome and systemic inflammatory responses: a randomised cross-over trial. Gut *68*, 1430–1438. https://doi.org/10.1136/gutjnl-2019-318424.
33. Wei, Z.h., Wang, H., Chen, X.y., Wang, B.s., Rong, Z.x., Wang, B.s., Su, B.h., and Chen, H.z. (2009). Time- and dose-dependent effect of psyllium on serum lipids in mild-to-moderate hypercholesterolemia: a meta-analysis of controlled clinical trials. Eur. J. Clin. Nutr. *63*, 821–827. https://doi.org/10.1038/ejcn.2008.49.
34. Gibb, R.D., McRorie, J.W., Jr., Russell, D.A., Hasselblad, V., and D'Alessio, D.A. (2015). Psyllium fiber improves glycemic control proportional to loss of glycemic control: a meta-analysis of data in euglycemic subjects, patients at risk of type 2 diabetes mellitus, and patients being treated for type 2 diabetes mellitus. Am. J. Clin. Nutr. *102*, 1604–1614. https://doi.org/10.3945/ajcn.115.106989.
35. Liu, X., Steiger, C., Lin, S., Parada, G.A., Liu, J., Chan, H.F., Yuk, H., Phan, N.V., Collins, J., Tamang, S., et al. (2019). Ingestible hydrogel device. Nat. Commun. *10*, 493. https://doi.org/10.1038/s41467-019-08355-2.
36. Raman, R., Hua, T., Gwynne, D., Collins, J., Tamang, S., Zhou, J., Esfandiary, T., Soares, V., Pajovic, S., Hayward, A., et al. (2020). Light-degradable hydrogels as dynamic triggers for gastrointestinal applications. Sci. Adv. *6*, eaay0065. https://doi.org/10.1126/sciadv.aay0065.
37. Bogdanski, P., Greenway, F., Miller, S., Matejkova, E., Mclean, B., Zmuda, W., Arciszewska, M., Sjödin, A.M., Salz, J., Mularczyk1, A.B., et al. (2022). Safety and Tolerability of Gelesis200 in the Management of Overweight and Obesity in People with Prediabetes or Type 2 Diabetes: Comprehensive Analysis of the LIGHT-UP Study ZoomForward2022: European Congress on Obesity (Obesity Facts).
38. Cani, P.D., Depommier, C., Derrien, M., Everard, A., and de Vos, W.M. (2022). Akkermansia muciniphila: paradigm for next-generation beneficial microorganisms. Nat. Rev. Gastroenterol. Hepatol. *19*, 625–637. https://doi.org/10.1038/s41575-022-00631-9.
39. Bourquin, L.D., Titgemeyer, E.C., Fahey, G.C., and Garleb, K.A. (1993). Fermentation of Dietary Fibre by Human Colonic Bacteria: Disappearance of, Short-Chain Fatty Acid Production from, and Potential Water-Holding Capacity of, Various Substrates. Scand. J. Gastroenterol. *28*, 249–255. https://doi.org/10.3109/00365529309096081.
40. Gill, S.K., Rossi, M., Bajka, B., and Whelan, K. (2021). Dietary fibre in gastrointestinal health and disease. Nat. Rev. Gastroenterol. Hepatol. *18*, 101–116. https://doi.org/10.1038/s41575-020-00375-4.

41. Marciani, L., Gowland, P.A., Spiller, R.C., Manoj, P., Moore, R.J., Young, P., and Fillery-Travis, A.J. (2001). Effect of meal viscosity and nutrients on satiety, intragastric dilution, and emptying assessed by MRI. Am. J. Physiol. Gastrointest. Liver Physiol. *280*, G1227–G1233. https://doi.org/10.1152/ajpgi.2001.280.6.G1227.

42. Stribiţcaia, E., Evans, C.E., Gibbons, C., Blundell, J., and Sarkar, A. (2020). Food texture influences on satiety: systematic review and meta-analysis. Sci. Rep. *10*, 1–18.

43. Cvijanovic, N., Isaacs, N.J., Rayner, C.K., Feinle-Bisset, C., Young, R.L., and Little, T.J. (2017). Lipid stimulation of fatty acid sensors in the human duodenum: relationship with gastrointestinal hormones, BMI and diet. Int. J. Obes. *41*, 233–239. https://doi.org/10.1038/ijo.2016.199.

44. Itoh, Y., Kawamata, Y., Harada, M., Kobayashi, M., Fujii, R., Fukusumi, S., Ogi, K., Hosoya, M., Tanaka, Y., Uejima, H., et al. (2003). Free fatty acids regulate insulin secretion from pancreatic β cells through GPR40. Nature *422*, 173–176.

45. Steneberg, P., Sykaras, A.G., Backlund, F., Straseviciene, J., Söderström, I., and Edlund, H. (2015). Hyperinsulinemia Enhances Hepatic Expression of the Fatty Acid Transporter Cd36 and Provokes Hepatosteatosis and Hepatic Insulin Resistance. J. Biol. Chem. *290*, 19034–19043. https://doi.org/10.1074/jbc.M115.640292.

46. Bisanz, J.E., Upadhyay, V., Turnbaugh, J.A., Ly, K., and Turnbaugh, P.J. (2019). Meta-Analysis Reveals Reproducible Gut Microbiome Alterations in Response to a High-Fat Diet. Cell Host Microbe *26*, 265–272.e4. https://doi.org/10.1016/j.chom.2019.06.013.

47. Depommier, C., Van Hul, M., Everard, A., Delzenne, N.M., De Vos, W.M., and Cani, P.D. (2020). Pasteurized Akkermansia muciniphila increases whole-body energy expenditure and fecal energy excretion in diet-induced obese mice. Gut Microb. *11*, 1231–1245. https://doi.org/10.1080/19490976.2020.1737307.

48. Yoon, H.S., Cho, C.H., Yun, M.S., Jang, S.J., You, H.J., Kim, J.-h., Han, D., Cha, K.H., Moon, S.H., Lee, K., et al. (2021). Akkermansia muciniphila secretes a glucagon-like peptide-1-inducing protein that improves glucose homeostasis and ameliorates metabolic disease in mice. Nat. Microbiol. *6*, 563–573.

49. Wilding, J.P.H., Batterham, R.L., Calanna, S., Davies, M., Van Gaal, L.F., Lingvay, I., McGowan, B.M., Rosenstock, J., Tran, M.T.D., Wadden, T.A., et al. (2021). Once-Weekly Semaglutide in Adults with Overweight or Obesity. N. Engl. J. Med. *384*, 989–1002. https://doi.org/10.1056/NEJMoa2032183.

50. Newsome, P.N., Buchholtz, K., Cusi, K., Linder, M., Okanoue, T., Ratziu, V., Sanyal, A.J., Sejling, A.S., and Harrison, S.A.; NN9931-4296 Investigators (2021). A Placebo-Controlled Trial of Subcutaneous Semaglutide in Nonalcoholic Steatohepatitis. N. Engl. J. Med. *384*, 1113–1124. https://doi.org/10.1056/NEJMoa2028395.

51. Jastreboff, A.M., Aronne, L.J., Ahmad, N.N., Wharton, S., Connery, L., Alves, B., Kiyosue, A., Zhang, S., Liu, B., Bunck, M.C., et al. (2022). Tirzepatide Once Weekly for the Treatment of Obesity. N. Engl. J. Med. *387*, 205–216. https://doi.org/10.1056/NEJMoa2206038.

52. DeFilipp, Z., Bloom, P.P., Torres Soto, M., Mansour, M.K., Sater, M.R.A., Huntley, M.H., Turbett, S., Chung, R.T., Chen, Y.-B., and Hohmann, E.L. (2019). Drug-Resistant E. coli Bacteremia Transmitted by Fecal Microbiota Transplant. N. Engl. J. Med. *381*, 2043–2050. https://doi.org/10.1056/NEJMoa1910437.

53. McCoubrey, L.E., Elbadawi, M., and Basit, A.W. (2022). Current clinical translation of microbiome medicines. Trends Pharmacol. Sci. *43*, 281–292. https://doi.org/10.1016/j.tips.2022.02.001.

54. Miele, L., Valenza, V., La Torre, G., Montalto, M., Cammarota, G., Ricci, R., Mascianà, R., Forgione, A., Gabrieli, M.L., Perotti, G., et al. (2009). Increased intestinal permeability and tight junction alterations in nonalcoholic fatty liver disease. Hepatology *49*, 1877–1887.

55. Thaiss, C.A., Levy, M., Grosheva, I., Zheng, D., Soffer, E., Blacher, E., Braverman, S., Tengeler, A.C., Barak, O., Elazar, M., et al. (2018). Hyperglycemia drives intestinal barrier dysfunction and risk for enteric infection. Science *359*, 1376–1383. https://doi.org/10.1126/science.aar3318.

56. Sannino, A., Ambrosio, L., Nicolais, L., and Demitri, C. (2007). Polymer Hydrogel and Preparing Method Thereof (Patent WO2007IT00584).

57. Sannino, A., Demitri, C., Zohar, Y., Hand, B.J., and Ron, E.S. (2012). Method for Producing Hydrogels (Patent WO2012170682A1).

58. Sannino, A., Demitri, C., Zohar, Y., Ron, E.S., Hand, B.J., and Saponaro, C. (2016). Method for Producing Hydrogels Coupling High Elastic Modulus and Absorbance (Patent WO2016123490A1).

59. Kleiner, D.E., Brunt, E.M., Van Natta, M., Behling, C., Contos, M.J., Cummings, O.W., Ferrell, L.D., Liu, Y.C., Torbenson, M.S., Unalp-Arida, A., et al. (2005). Design and validation of a histological scoring system for nonalcoholic fatty liver disease. Hepatology *41*, 1313–1321. https://doi.org/10.1002/hep.20701.

60. Galarraga, M., Campión, J., Muñoz-Barrutia, A., Boqué, N., Moreno, H., Martínez, J.A., Milagro, F., and Ortiz-de-Solórzano, C. (2012). Adiposoft: automated software for the analysis of white adipose tissue cellularity in histological sections. J. Lipid Res. *53*, 2791–2796. https://doi.org/10.1194/jlr.D023788.

61. Furet, J.P., Firmesse, O., Gourmelon, M., Bridonneau, C., Tap, J., Mondot, S., Doré, J., and Corthier, G. (2009). Comparative assessment of human and farm animal faecal microbiota using real-time quantitative PCR. FEMS Microbiol. Ecol. *68*, 351–362. https://doi.org/10.1111/j.1574-6941.2009.00671.x.

62. Bolger, A.M., Lohse, M., and Usadel, B. (2014). Trimmomatic: a flexible trimmer for Illumina sequence data. Bioinformatics *30*, 2114–2120. https://doi.org/10.1093/bioinformatics/btu170.

63. Langmead, B., and Salzberg, S.L. (2012). Fast gapped-read alignment with Bowtie 2. Nat. Methods *9*, 357–359. https://doi.org/10.1038/nmeth.1923.

64. Beghini, F., McIver, L.J., Blanco-Míguez, A., Dubois, L., Asnicar, F., Maharjan, S., Mailyan, A., Manghi, P., Scholz, M., Thomas, A.M., et al. (2021). Integrating taxonomic, functional, and strain-level profiling of diverse microbial communities with bioBakery 3. Elife *10*, e65088. https://doi.org/10.7554/eLife.65088.

65. Bolyen, E., Rideout, J.R., Dillon, M.R., Bokulich, N.A., Abnet, C.C., Al-Ghalith, G.A., Alexander, H., Alm, E.J., Arumugam, M., Asnicar, F., et al. (2019). Reproducible, interactive, scalable and extensible microbiome data science using QIIME 2. Nat. Biotechnol. *37*, 852–857. https://doi.org/10.1038/s41587-019-0209-9.

66. Wirbel, J., Pyl, P.T., Kartal, E., Zych, K., Kashani, A., Milanese, A., Fleck, J.S., Voigt, A.Y., Palleja, A., Ponnudurai, R., et al. (2019). Meta-analysis of fecal metagenomes reveals global microbial signatures that are specific for colorectal cancer. Nat. Med. *25*, 679–689. https://doi.org/10.1038/s41591-019-0406-6.

67. Letunic, I., and Bork, P. (2021). Interactive Tree Of Life (iTOL) v5: an online tool for phylogenetic tree display and annotation. Nucleic Acids Res. *49*, W293–W296. https://doi.org/10.1093/nar/gkab301.

STAR★METHODS

KEY RESOURCES TABLE

REAGENT or RESOURCE	SOURCE	IDENTIFIER
Antibodies		
Mouse monoclonal anti-CD34	eBioscience	Clone RAM34; cat.14-0341-82; RRID: AB_467210
Mouse monoclonal anti-ZO-1	Invitrogen	ZO1-1A12; cat.33-9100; RRID: AB_2533147
Rabbit monoclonal anti-MUC2	Abcam	EPR23479-47; cat.ab272692; RRID: AB_2888616
Rat monoclonal anti-E-Cadherin	Abcam	Clone DECMA-1; cat.ab11512; RRID: AB_298118
Mouse monoclonal anti-Insulin	BD-Pharmingen	Clone T56-706; cat.565688; RRID: AB_2739330
Mouse monoclonal anti-Glucagon	BD-Pharmingen	Clone U16-850 RUO; cat.565860; RRID: AB_2739382
Rabbit monoclonal anti-CD3	Invitrogen	SP7; cat.MA1-90582; RRID: AB_1956722
Rat monoclonal anti-F4/80	Invitrogen	BM8; cat.MF48000; RRID: AB_10376289
Rat monoclonal anti-B220	eBioscience	RA3-6B2; cat.14-0452-82; RRID: AB_467254
Rat-on-Mouse HRP-Polymer	Biocare Medical	RT517G; cat.RT517
Rat anti- mouse CD16/CD32	eBioscience	Clone 93; cat.16-0161-85; RRID: AB_468899
Hamster anti-mouse CD3 APC	BD Biosciences	Clone 145-2c11; cat.561826; RRID: AB_10896663
Rat anti-mouse CD11b 421	BD Biosciences	Clone M1-70; cat.562605; RRID: AB_11152949
Rat anti-mouse Ly6C FITC	BD Biosciences	Clone AL-21; cat.553104; RRID: AB_394628
Rat anti-mouse Ly6G BV510	BD Biosciences	Clone 1A8; cat.551461; RRID: AB_394208
Hamster anti-mouse CD11c A700	BD Biosciences	Clone HL3; cat.560583; RRID: AB_1727421
Hamster anti-mouse CD103 PE	Biolegend	Clone 2E7; cat.121406; RRID: AB_1133989
Rat anti-mouse MHC II PerCp 5.5	Biolegend	clone M5-114-15.2; cat.107625; RRID: AB_2191072
Mouse anti-mouse NK 1.1 APC eFluor 780	Invitrogen	clone PK136; cat. 47-5941-82; RRID: AB_2735070
Fixable Viability Dye 780	BD Biosciences	cat. 565388; RRID: AB_2869673
Bacterial and virus strains		
A. muciniphila	DSMZ-German Collection of Microorganisms and Cell Cultures GmbH	ATCC-BAA-835
Chemicals, peptides, and recombinant proteins		
Betazoid DAB chromogen kit	Biocare Medical	Cat.BDB2004
Ampicillin	GoldBio	Cat.A-301-100
Vancomycin	GoldBio	Cat.V-200-25
Neomycin	GoldBio	Cat.N-620-100
Metronidazole	MPBiomedicals	Cat.443-48-1
Porcine mucin type III	Sigma	Cat.M1778
Anaerobe basal broth	OXOID	Cat.CM0957B
D-Glucose	Sigma	Cat.158968
Diva Decloaker 1X	Biocare Medical	Cat.DV2005L2J
Rodent Block M	Biocare Medical	Cat.RBM861
Carmine	Sigma	Cat.C1022
Methylcellulose	Sigma-Aldrich	Cat.M0512
Oil Red O	Sigma-Aldrich	Cat.102419
Triton X-100	Sigma Aldrich	Cat.9036
Betazoid DAB Chromogen Kit	Biocare Medical	cat. BDB2004
Rat on Mouse HRP Polymer	Biocare Medical	cat. RT517G
FITC-labelled Dextran 4 kDa	TdB Labs	Cat. 60842-46-8
Critical commercial assays		
Mouse Ultrasensitive Insulin ELISA	Mercodia AB	10-1249-01

(*Continued on next page*)

Continued

REAGENT or RESOURCE	SOURCE	IDENTIFIER
Mouse Glucagon-Like Peptide 1 ELISA	MyBioSource	MBS260820
Chromogenic Endotoxin Quantitation Kit	ThermoScientific	A39552
TGs Colorimetric Assay KIT	CaymanChem	CAY-10010303-96
Low-density Lipoprotein Cholesterol (LDL-C) Colorimetric Assay Kit	Elabscience	E-BC-K205
High-density Lipoprotein Cholesterol (HDL-C) Colorimetric Assay Kit	Elabscience	E-BC-K221
ALT Color Microwell	Sentinel diagnostics	17238A
ImProm-II Reverse Transcriptase kit	Promega	A3801
Quick-RNA MiniPrep Kit	Zymo Research	R1055
Fast SYBR Green Master Mix	ThermoFisher	4385612
G'NOME DNA isolation kit	MPBiomedicals	112010400-CF
**Deposited data**		
Metagenome-sequencing data	Sequence Read Archive [SRA]:PRJNA1010707	https://www.ncbi.nlm.nih.gov/bioproject/PRJNA1010707
**Experimental models: Organisms/strains**		
Mouse: C57BL/6J male 8 weeks old	Charles River Laboratories	
**Oligonucleotides**		
Please refer to Table S2		
**Software and algorithms**		
Prism 8	GraphPad Prism	https://www.graphpad.com; RRID:SCR_002798
QuPath		https://qupath.github.io/; RRID: SCR_018257
Fiji	ImageJ	https://imagej.net/; RRID:SCR_002285
Biorender		https://www.biorender.com
FlowJo	FlowJo	https://www.bdbiosciences.com/en-us/products/software/flowjo-v10-software
Trimmomatic v0.39	Bioweb	http://www.usadellab.org/cms/index.php?page=trimmomatic
MetaPhlAn v3.0.14		https://anaconda.org/bioconda/metaphlan
Qiime2 v2022.2		
ITOL web tool		https://itol.embl.de/
Humann v3.0.1		https://huttenhower.sph.harvard.edu/humann/

RESOURCE AVAILABILITY

Lead contact

Further information and requests for resources and reagents should be directed to and will be fulfilled by the lead contact, Maria Rescigno (maria.rescigno@hunimed.eu).

Materials availability

This study did not generate new unique reagents.

The OSH used in this study is property of Gelesis LLC. The OSH will be made available on request, but Gelesis may require a payment and/or a completed Material Transfer Agreement.

Data and code availability

- Raw data reported in this paper will be shared by the lead contact upon request.
- Metagenome-sequencing data have been deposited at SRA and are publicly available as of the date of publication. Accession numbers are listed in the key resources table.
- Any additional information required to reanalyze the data reported in this paper is available from the lead contact upon request.

EXPERIMENTAL MODEL AND STUDY PARTICIPANT DETAILS

Animals

C57BL/6J male, 8 weeks old mice were purchased from Charles River Laboratories and maintained on a 12 hours light-dark cycle with controlled temperature and under specific pathogen-free conditions. Mice were housed under specific-pathogen-free (SPF) conditions at Campus IFOM-IEO (Milan, Italy) and Humanitas Clinical and Research Center (Rozzano, Milan, Italy) and maintained on a 12 hours light-dark cycle. Experiments were performed following the guidelines established in the Principles of Laboratory Animal Care (directive 2010/63/EU) and approved by the Italian Ministry of Health. All animal experiments were performed under protocols (31/2013, 139/2015-PR, 532/2017-PR, 598/2019-PR, 11/2023-PR, 757/2023-PR) approved by the Italian Ministry of Health, and consistent with national (D.L. N. 26, G.U. March 4, 2014) and international law and policies (EEC Council Directive 2010/63/EU). Investigators were not blinded during experimental mice allocation and outcome assessment, except for histological scoring. To induce obesity, mice were fed a High-Fat diet (HFD) (PF1916, TD.06415 Envigo, Mucedola, Italy; 4.59 Kcal/g, 45% from fat, lard 195 g/Kg with a fatty acid profile: 36% saturated, 47% monounsaturated). For the NASH setting, we used a High-Fat, High-Cholesterol and Carbohydrates (HFHCC) diet [5TJT, TestDiet, USA; 4.49 Kcal/g, 40% from fat, 1% cholesterol plus 42 g/L of a mixture of fructose and D-glucose (55:45 w/w) in the drinking water] or a Methionine-choline-deficient diet (MCDD) (TD.90262, Envigo). The experimental models were subjected to treatment with food supplemented with either 2% or 4% OSH following different regimens. Control mice were fed either a low-fat diet (12.6% fat, 67.3% CHO; 3.7 Kcal/g, TD.86489, Envigo) or a chow diet (9% fat, 3.87 Kcal/g; 4RF21, Mucedola, Italy) (Table S1). Fecal pellets were collected at different timepoints and snap-frozen until analyzed. Animals were euthanized at different time points by CO_2 inhalation, with terminal collection of blood by cardiac puncture; liver, gut, and adipose tissue were precisely excised, measured and stored at -80 °C or fixed. Animal weight and cage observations were routinely checked after the introduction of the diet; no gross changes in behavior or any other symptoms were noted with any intervention (diet/treatment/procedure). Cumulative food intake was calculated on the basis of the first 4-8 weeks of treatment. Energy efficiency calculation corresponds to the ratio of body weight gain and energy intake (Kcal/g).

To assess the effect of OSH on *Akkermansia* repopulation in gut microbiota, we first depleted the gut microbiota by using a combination of broad-spectrum antibiotics for 1 week [ampicillin sodium (1 g/L, A-301-100, GoldBio), vancomycin hydrochloride (0.5 g/L, V-200-25, GoldBio) and neomycin sulfate (1 g/L, N-620-100, GoldBio) in the drinking water plus metronidazole (2 mg/mouse, 443-48-1, MPBiomedicals) every other day via gavage]. After antibiotic treatment, mice were randomly divided into receiving a chow diet (4RF21, Mucedola, Italy) with or without 4% of OSH for two further weeks. Fecal pellets were collected at different time points and snap-frozen until used.

Microbe strain

A. muciniphila (ATCC-BAA-835) was purchased from the Leibniz Institute DSMZ-German Collection of Microorganisms and Cell Cultures GmbH and cultivated into liquid anaerobe basal medium (OXOID, ThermoFisher), supplemented with 0.05% porcine mucin type III (Sigma) and 4 g/L D-Glucose (Sigma) in an anaerobic workstation (Baker Ruskinn) at 37°C. The concentration of OSH, its precursors, and fibers to test was calculated based on the medium uptake ratio (MUR) of the OSH in these conditions, being that able of occupying half the volume of the culture tube when the hydrogel was fully hydrated in steady state (5 g/L). MUR was determined as described previously[15]. All the experiments included a control tube with half the volume of the medium to avoid any concentration bias due to the absorption capacity of the OSH.

METHOD DETAILS

OSH synthesis and diet preparation

OSH was synthesized from carboxymethylcellulose sodium salt (CMC) and citric acid (CA), via a proprietary technological platform described elsewhere.[56–58]

Based on the findings of a recent study,[15] a CMC of high molecular weight (2.5 x 10^6 Da) was used to obtain an OSH formulation with optimized elasticity, found to preserve intestinal homeostasis and tissue integrity in an *ex-vivo* organ culture model (8). Briefly, we prepared a mixture of CMC and citric acid (CA/CMC weight ratio=0.002) in purified water through planetary mixing. Once homogeneous, the mixture was dried, ground, sieved and crosslinked at 120 °C for 4 h. Then, the powder was washed, filtered, and again dried, ground, and sieved to achieve a final particle size of 100-1000 μm. The resulting OSH (GS200) shows an average medium uptake ratio (MUR) in simulated gastric fluid/water (1:8) of 77 g/g, while the elasticity in the same medium (expressed in terms of elastic modulus G') is about 1800 Pa. The OSH described in this work, shows a solid-like mechanical behavior, with elasticity that is typically one order of magnitude higher than viscosity.

In order to prepare diet formulations including a given percentage of OSH (either 2% or 4%), as per the experimental design discussed above, the dry hydrogel powder was directly added to the given diet during the manufacturing, taking care to avoid the addition of water to prevent any OSH hydration. To ensure consistency and maintain a constant caloric value between diets with and without OSH, the cellulose content of the diets (~5%) was replaced partially or entirely with the hydrogel quantity added in the preparation.

Histopathological assessment

Tissues were fixed with 10% formaldehyde or Periodate-Lysine-Paraformaldehyde (PLP) buffer overnight. Hematoxylin and eosin staining was performed in paraffin-embedded formalin-fixed sections to assess the morphology of the liver, intestine, and adipose tissue. The extent of hepatic lesions was evaluated by two expert examiners in a blinded fashion according to the NAFLD activity score (NAS),[59] which is based on the combination of features observed histologically, and the score is calculated by adding together the scores for each of the individual features with higher scores indicating more severe disease: A) Steatosis (percentage in liver): 0-3 points (0=less than 5%, 1=5-33%, 2=33-66%, 3=more than 66% B) Lobular inflammation: 0-3 points (0=none, 1=less than 2 foci per 200x field, 2=2-4 foci per 200x field, 3=more than 4 foci per 200x field) C) Hepatocyte ballooning: 0-2 points (0=none, 1=few ballooned cells, 2=many ballooned cells). Each score is the average of 10 fields per sample.

Liver fibrosis was assessed by Sirius red (Sigma-Aldrich, USA) staining, calculating the area with the software QuPath (https://qupath.github.io). Adipocytes area was calculated using the Image J software with the plugin Adiposoft.[60] Oil red O (ORO) (Sigma-Aldrich, USA) staining was performed on PLP buffer-fixed OCT-embedded frozen liver sections according to the manufacturer's protocol to observe lipid droplets in liver tissues. Bright-field images were acquired with a virtual slide microscope VS120 (Olympus) using a dry objective with x10 or x20 magnification.

Intestinal epithelial ZO-1 was quantified in PLP buffer-fixed OCT-embedded intestinal samples by immunofluorescence. Cryosections (10 μm thick) were rehydrated, blocked with 0.1 Tris-HCl pH 7.4, 2% FBS, 0.3% Triton X-100 and stained with an anti-mouse ZO-1 (clone ZO1-1A12, Invitrogen). In order to avoid endothelial ZO-1 expression, an anti-mouse CD34 (clone RAM34, eBioscience) was used to create a virtual mask for the image analysis and quantification using the software Fiji (Image J). MUC-2 was quantified by immunofluorescence in intestinal samples; 12 μm thick cryosections were rehydrated in PBS with Tween20 (0.05%), antigen retrieval was performed using sodium citrate pH 6 for 20 minutes, blocked with 0.1 Tris-HCl pH 7.4, 2% FBS, 0.3% Triton X-100 and stained with an anti-MUC-2 (rabbit monoclonal anti-MUC-2 EPR23479-47, ab272692 Abcam). Anti-E-Cadherin antibody (clone DECMA-1, Abcam) was used to encircle intestinal epithelial cells.

Pancreatic insulin was quantified in PLP buffer-fixed OCT-embedded intestinal samples by immunofluorescence in pancreas tissue samples. 6 μm thick cryo-sections were rehydrated, blocked with 0.1 Tris-HCl pH 7.4, 2% FBS, 0.3% Triton X-100 and stained with an anti-insulin (clone T56-706, BD Pharmingen) and anti-glucagon (clone U16-850 RUO, BD Pharmingen). Tissues were incubated with the appropriate fluorescent secondary antibody and, before imaging, nuclei were counterstained with DAPI. Acquisition by confocal microscopy was performed on a Leica SP8 (oil objective) at x40 - x63 magnification.

Immunohistochemical analysis of liver CD3, F4/80, B220 and Ly6G markers was performed on 3 μm-thick tissue sections prepared from FFPE mouse liver samples. Following deparaffinization and re-hydration steps, antigen retrieval was performed with 1mM EDTA (pH 8) at 98°C in a water bath for CD3 and B220 and with heat retrieval solution Diva Decloaker 1X (cat. DV2005L2J, Biocare Medical) for F4/80 and in a pressure cooker for 20 minutes for Ly6G. Endogenous peroxidases were then quenched with 3 % H_2O_2. Slides were blocked with Rodent Block M (cat. RBM961, Biocare Medical) for 1 hour in a humidified chamber. Slides were incubated for 2 hours with primary antibodies against CD3 (monoclonal cat. MA1-90582HPA002279, Invitrogen), F4/80 (monoclonal cat. MF48000, Thermo Fisher Scientific), B220 (clone RA3-6B2, eBioscience) in PBS supplemented with 0.1% BSA. Rat on Mouse HRP Polymer (cat. RT517G, Biocare Medical) was used as secondary antibody. After washing, slides were developed with DAB (3,30-diaminobenzidine) (Betazoid DAB Chromogen Kit, cat. BDB2004; Biocare Medical) and counterstained with Hematoxylin. Tissues were dehydrated with ethanol, mounted with Eukitt, and acquired with an Olympus BX51widefield microscope, 20-40x objective.

FITC-dextran permeability assay

In order to assess gut barrier permeability, mice were starved in the morning for 4 hours and then orally administered through gavage 400 mg/Kg of FITC-labelled dextran of 4 kDa (TdB Labs). Blood was collected from the tail vein after 4 hours and the concentration of FITC-Dextran in plasma was quantified by fluorescence intensity against a standard curve (Clariostar Plus; BMG Labtech, USA).

Glucose tolerance and Insulin Tolerance Tests

After 6-hours of fasting, basal glycemia was measured collecting blood from the tip of the tail vein using a hand-held whole-blood glucose monitor (Accu-Chek, Roche, Switzerland). Subsequently, a bolus of glucose (GTT; 2 g/kg mouse, Sigma) or insulin (ITT; 0.2 UI/kg mouse, Humulin R, Lilly) was intraperitoneally injected; blood glucose levels were then recorded every 30 minutes for 2 hours in both tests. Glucose Area Under the Curve (AUC; 0-120 minutes) was determined. The Homeostatic Model Assessment of Insulin Resistance (HOMA-IR) was calculated as (Fasting insulin) * (Fasting glucose)/X; X was derived from mean glucose and insulin levels in the experiment.

Carmine red dye gut transit time assay

Whole gastric time was quantified administering a 6% carmine (C1022, Sigma-Aldrich) solution in 0,5% methylcellulose (M0512, Sigma-Aldrich) dissolved in distilled water, via oral gavage and recording time of passage in the fecal stream. In detail, each mouse received a *gavage* with 200 μL of 6% carmine solution and time of administration was recorded for each individual mouse. Given that the whole mouse gut transit time typically > 2 hours, observation started 90 minutes after carmine solution administration. Each mouse was transferred in an individual cage until a red fecal pellet was produced. Time of expulsion of the red fecal pellet was

recorded and the mouse was returned to its original cage immediately after the production of the red fecal pellet. Whole gastric time was calculated as the time interval between the gavage and the first observed red fecal pellet. Importantly we did not squeeze or apply pressure to the mouse abdomen to stimulate passage of fecal pellets, as stress also can influence gut transit.

Biochemical analysis in serum/plasma

Fasting levels of insulin and GLP-1 were assessed in plasma EDTA collected from the tip of the tail vein at baseline from either GTT or ITT assays in EDTA-coated tubes. Plasma was retrieved by centrifugation and stored at -20°C until assayed. Insulin and GLP-1 levels were evaluated by ELISA kits (Mouse Ultrasensitive Insulin ELISA, Mercodia AB, and Mouse Glucagon-Like Peptide 1 ELISA Kit, MyBioSource). All other circulating parameters were assessed on terminal serum collected through heart puncture at sacrifice. Serum was retrieved by centrifugation and stored at -20°C until assayed. LPS was measured by the Limulus Amebocyte Lysate assay (Pierce), and triglycerides by TGs Colorimetric Assay KIT (CAY-10010303-96, CaymanChem). Low-Density Lipoprotein Cholesterol (LDL-C) and High-Density Lipoprotein Cholesterol (HDL-C) were measured through colorimetric assay kits (E-BC-K205 and E-BC-K221, respectively from Elabscience), while total cholesterol was calculated crossing HDL-C, LDL-C and TGs values of each individual mouse (applying Friedewald's formula). Alanine aminotransferase levels were measured through colorimetric tests (ALT color Microwell, Sentinel Diagnostics). All the analyses were performed following manufacturers' instructions.

Hepatic triglycerides quantification

Frozen liver tissue was homogenized in NP-40 substitute reagent at a concentration of 50 mg/mL, after adding protease inhibitors, with the help of a TissueRuptor II (Qiagen, Germany). Samples were heated to 80°C for 10 minutes and then centrifuged at 10,000g for 10 minutes. Total hepatic triglyceride content, expressed as mg/g tissue was measured in the supernatant by employing the TGs Colorimetric Assay Kit (CaymanChem, USA).

Quantification of DNA and mRNA by real-time PCR

Frozen liver or intestine tissues (25-50mg) were placed in 2 ml microcentrifuge tubes with stainless steel beads and homogenized with 500 μL of TRIzol (Invitrogen) for 2 cycles of 2 minutes at 30 Hz in a TissueLyser II (Qiagen). RNA was extracted by adding 100 μL of chloroform, precipitating the aqueous phase with 1 volume of 100% ethanol, and purified using Quick-RNA MiniPrep Kit (Zymo Research) following the manufacturer's instructions. Reverse transcription of 1 μg of RNA was done using the ImProm-II Reverse Transcriptase kit (Promega) with random primers (Invitrogen).

Real Time-PCR assay was performed with Fast SYBR Green Master Mix (Life Technologies) using 2.5 ng DNA or cDNA equivalents per reaction. Primer sequences are listed in Table S2. Gene expression levels were normalized to the expression of *Rpl32* or *18S rRNA* and quantified using the $2^{-\Delta\Delta Ct}$ method.

To check for the abundance of *A. muciniphila,* we extracted bacterial DNA from fecal samples using the protocol described below, and then we quantified the presence of *A. muciniphila* using strain-specific primers, normalizing with the total *16S rRNA* levels.

Flow cytometry

Mice were euthanized and livers were weighed and harvested in complete medium (DMEM, 10% fetal bovine serum North American, 1% Penicillin/Streptomycin and 1% Glutamine). Livers were minced and enzymatically digested for 30 minutes at 37°C in digestion buffer (DMEM, 10% FBS, 1% Glu, 1% P/S with 1 mg/mL Collagenase D (Roche) and 5 U/mL DNAse (Roche). Digestion process was stopped adding cold FACS buffer solution (PBS, 10% FBS, 0,1% NaN_3 2 mM EDTA) to each sample. The digested mixture was then transferred onto a 70μm cell strainer and any undigested liver piece was mashed using the end of a syringe plunger. The strained cell suspension was centrifuged at 50 x g for 3 minutes. Supernatant containing non-parenchymal cells (NPCs), was collected and centrifuged at 167 g for 10 minutes. And pellet containing NPCs was resuspended in 40% Percoll and stratified on an 80% Percoll solution to separate mononuclear cells from hepatocytes and stromal cells through centrifugation for 20 minutes at 167 g and red erythrocytes were then eliminated through incubation with e-Lysis buffer. Cells were then resuspended in FACS buffer solution and surface-labeled. The immune cells were incubated with anti-CD16/CD32 antibody (clone 93, cat.16-0161-85, eBioscience) and stained with the following antibodies: anti-mouse CD45.2 PeCy7 (clone 104, BD Biosciences), anti-mouse CD3 APC (clone 145-2c11, BD Biosciences), anti-mouse CD11b 421 (clone M1-70, BD Biosciences), anti-mouse Ly6C FITC (clone AL-21, BD Biosciences), anti-mouse Ly6G BV510 (clone 1A8, BD Biosciences), anti-mouse CD11c A700 (clone HL3, BD Biosciences), anti-mouse CD103 PE (clone 2E7, Biolegend), anti-mouse MHC II PerCp 5.5 (clone M5-114-152, Biolegend) and anti-mouse NK 1.1 APC eFluor 780 (clone PK136, Invitrogen). Dead cells were excluded by using Fixable Viability Dye 780 (cat. 565388, BD Biosciences, 1:1000). Flow cytometry was performed on Fortessa (BD Biosciences) platform and results were analyzed using FlowJo software version 10.5.3.

Fecal microbiota transplantation

Fecal pellets were collected and crushed on a 70 μm filter with sterile PBS. Samples were centrifuged at 500g for 3 minutes to remove debris (i.e., fibers and eventual hydrogel particles). A total volume of 200 μL from the bacteria-enriched supernatant was then gavaged at 100 mg/mL. Donor animals were fed an HFHCC diet for 10 weeks and then randomly divided into receiving HFHCC, or HFHCC with 2% or 4% OSH. After 2 weeks of treatment, fecal pellets were freshly collected every other day and transplanted to Recipient animals for a total period of 4 weeks. Recipients were fed an HFHCC and run in parallel to the Donors and for the same period.

Gut metagenomic analysis

DNA extraction

DNA from fecal pellets was extracted with G'NOME DNA isolation kit (MP) following a published protocol.[61] Briefly, fecal pellets, stored at -80 °C, were homogenized in 550 μL Cell Suspension Solution. After the addition of 50 μL RNase Mix and 100 μL Cell Lysis/Denaturing Solution samples were incubated at 55°C for 30 minutes. Samples were then incubated for further 2 hours at 55°C after adding 25 μL Protease Mix. Samples then underwent mechanical disruption of bacterial cells with 0.1 mm zirconia/silica beads (BioSpec) in FastPrep-24 homogenizer (MP Biomedicals). Lysates were retrieved. Beads were washed three times with 400 μL of TENP buffer (50 mM Tris pH 8, 20 mM EDTA pH 8, 100 mM NaCl, 1% PVPP). Supernatants were pooled with the original lysate and precipitated with isopropanol. DNA pellet was resuspended in 400 μL water and incubated with 100 μl of Salt Out Mixture to remove impurities. Samples were then precipitated in 100% ethanol and the DNA pellet was washed with 70% ethanol. DNA pellets were dried and resuspended in water. Concentrations of extracted DNA from each sample were determined by Qubit measurement and an estimate of sample purity was determined with spectrophotometry by measuring the A260/A280 absorbance ratio.

DNA sequencing

Libraries were prepared using the Nextera XT Library Prep (Illumina) in which sample DNA is simultaneously enzymatically fragmented and tagged with primer sites for adapter/index addition. Sequencing adapters and indices were added during PCR amplification of the fragmented DNA. Fragment analysis on the Agilent Bioanalyzer was performed on a random selection of the samples to verify library size. Paired-end shotgun sequencing (2 x 150 bp) of the prepared libraries was performed on the Illumina NextSeq. Sequencing run and data quality were evaluated using the following metrics: Q-score > Q30, cluster density, clusters passing filter, sequencing yield, and PhiX alignment percentage. Whole metagenome sequencing was performed to a target depth of at least 10 million paired-end reads per sample on Illumina's NextSeq platform at DNA Genotek Inc. and CosmosID facilities.

Bioinformatic analysis

Quality filtering of sequencing reads was executed with Trimmomatic v0.39[62] using the following parameter: MINLEN:100 AVGQUAL:30. Alignment to the mouse reference genome (GRCm39) was performed with Bowtie2 v2.4.5[63] in order to remove host-derived sequences. High-quality and clean reads were analyzed with MetaPhlAn v3.0.14[64] in order to profile the composition of the microbial communities at species-level resolution. Relative abundance tables were converted into pseudo-counts and used to perform alpha and beta diversity analysis with Qiime2 v2022.2.[65] Alpha diversity was evaluated by Chao1 and Shannon index and represented by the box-and-whisker plot. Community dissimilarities (β-diversity) were evaluated by Jaccard index and represented by a principal coordinate analysis plot. Differences in alpha-diversity indices across experimental groups were evaluated with the Wilcoxon rank-sum test. The qiime diversity beta-group-significance function was used to assess differences in the microbiome composition across the different experimental groups with permutational multivariate analysis of variance (PERMANOVA). Bacteria showing different abundance between experimental groups were identified using the Wilcoxon rank-sum test (FDR <0.05). Bacteria phyla showing different abundance between experimental groups were identified using one-way ANOVA, Dunnett's multiple comparison test. The magnitude of the change in abundance between conditions was expressed as a generalized fold change as described in Wirbel et al. 2019.[66] A cladogram displaying the phylogenetic relationship of the bacteria was created with the ITOL web tool.[67] Humann v3.0.1[64] was used to profile the abundance of sequences of microbial metabolic pathways and carbohydrate-active enzymes in the mouse metagenome, whose functions were checked via www.cazy.org, using a threshold of FDR<0.05. To unravel common species or pathways in both settings, preventative and therapeutic, we filtered for those with FDR<0.1 in independent 2% and 4% OSH vs HFD comparisons and then selected only common hits with a gFC>|0.3|.

QUANTIFICATION AND STATISTICAL ANALYSIS

Statistical analysis was performed using GraphPad PRISM (v.9.0, GraphPad Software, USA). Values were compared using either a Student's t-test for single variable or one-way ANOVA and two-way ANOVA with Dunnett's multiple comparison test depending on the distribution of the data. Chi-square test was used for categorical data. Outliers were identified by using Grubb's test at an alpha=0.05 and removed, where appropriate. Results are represented as Mean ± SEM for ≥ 3 samples or independent experiments; the exact number is indicated in the figure legends. *p<0.05, **p<0.01, ***p<0.001, ****p<0.0001.

Cell Reports Medicine, Volume *4*

Supplemental information

Biomimetic superabsorbent hydrogel acts as a gut protective dynamic exoskeleton improving metabolic parameters and expanding *A. muciniphila*

Alessandra Silvestri, Antonio Gil-Gomez, Milena Vitale, Daniele Braga, Christian Demitri, Paola Brescia, Marta Madaghiele, Ilaria Spadoni, Bryan Jones, Giulia Fornasa, Juliette Mouries, Sara Carloni, Michela Lizier, Manuel Romero-Gomez, Giuseppe Penna, Alessandro Sannino, and Maria Rescigno

SUPPLEMENTARY MATERIAL

Figure S1

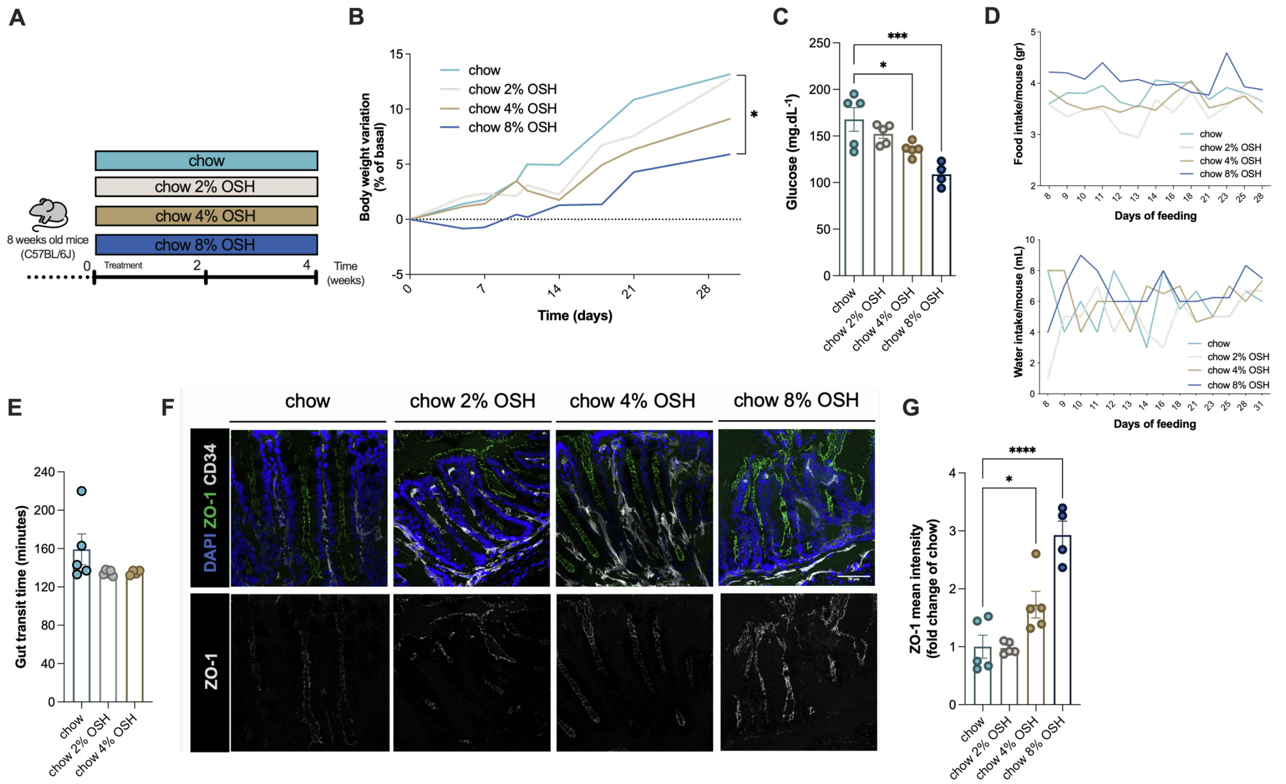

***Supplementary Figure 1*. Selection of OSH dose. Related to Figure 1, 3 and 4.**
a. Experimental scheme: 8 weeks old C57BL/6J male mice were fed for 4 weeks with: chow diet (light blue) and chow diet supplemented with increasing concentrations of OSH (2 - 4 and 8 % respectively gray, brown and blue). **b.** body weight variation expressed as percentage of basal body weight; **c.** fasting blood glucose levels, measured after 4 weeks of feeding with OSH supplemented chow diet; **d.** food intake expressed as food intake in grams per day per mouse; cumulative water intake expressed as water intake in mL per day per mouse; **e.** gut transit time after 1 week OSH treatment; **f.** colon tissue sections of mice fed for 4 weeks with OSH supplemented chow diet stained for ZO-1 (green), CD34 (gray) and DAPI (blue). First line is depicting the three merged channels. Second line is illustrating images of ZO-1 channel alone (gray). Representative images of a single mouse out of 5 mice per group. Scale bar 50 µm; **g.** quantification of ZO-1 fluorescent signal expressed as fold change of control diet. (*p<0.05; **p<0.01; ***p<0.001; **** <0.0001 one-way ANOVA Dunnett's post-test, line at mean with SEM).

Figure S2

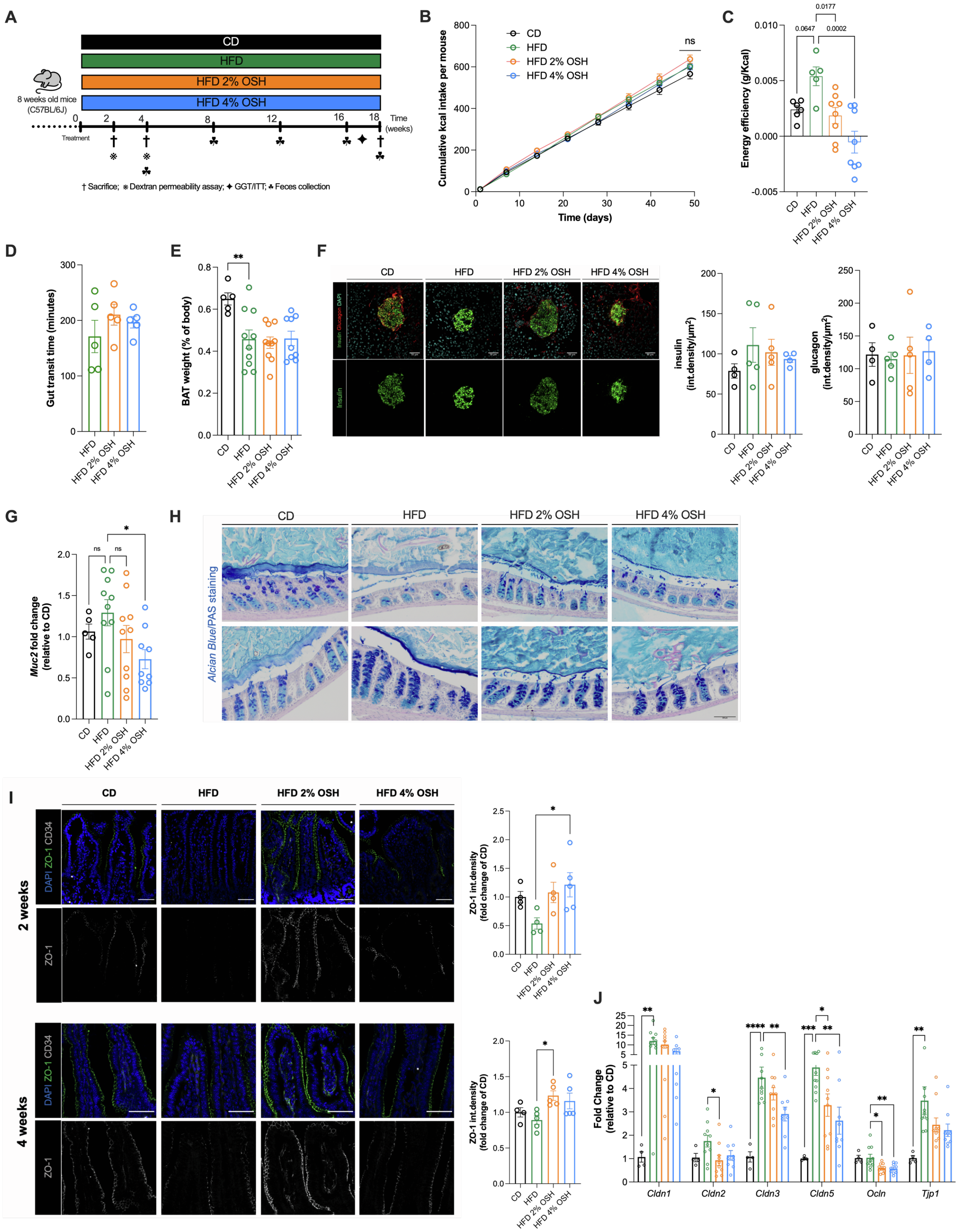

***Supplementary Figure 2*. Effects of preventative OSH feeding. Related to Figures 1 and 2.**
C57BL/6J male mice at 8 weeks of age were fed for 18 weeks with: control diet (CD, black) and HFD (green), HFD 2% OSH (orange) and HFD 4% OSH (light blue); after 2- and 4-weeks mice were subjected to 4kDa FITC-Dextran permeability assay and sacrificed to assess intestinal barrier status. Fecal samples were collected after 4,8,12,16 weeks of feeding and at sacrifice for gut microbiota analysis. Glucose Tolerance Test (GTT) and Insulin Tolerance Test (ITT) were performed after 17 weeks of feeding. **b**. Kilocalorie intake, expressed as cumulative kcal intake per mouse, in time (days); **c.** Energy efficiency, calculated as the weight gained over 28 days divided by the calories absorbed over the 28 days for each mouse; **d.** gut transit time after 1 week OSH

treatment; **e.** brown adipose tissue (BAT) weight after 18 weeks of feeding, expressed as percentage of body weight; **f.** pancreatic tissue sections of mice fed for 18 weeks, stained for glucagon (red), insulin (green) and DAPI (cyan). First line is depicting the three merged channels. Second line is illustrating images of insulin channel alone (gray). Representative images of a single mouse out of 4-5 mice per group. Scale bar 50 µm. On the side, quantification of insulin and glucagon fluorescent signal expressed as integrated density per μm^2, analyzed using Fiji image software; **g**. Relative *Muc2* gene expression levels in intestinal tissue samples after 18 weeks of OSH treatment expressed as fold change of CD fed mice (n=5 mice per group CD, n=10 mice per group HFD and 2% OSH, n=9 mice per group HFD 4% OSH) (*p<0.05; one-way ANOVA Dunnett's post-test, line at mean with SEM); **h.** Alcian Blue/PAS staining of *Carnoy's* fixed PE colon tissue sections of mice treated for 18 weeks with OSH supplemented diet. Representative images of two mice out of 9/10 animals per group. Scale bar 100 µm; **i.** ileum tissue sections of mice fed for 2 and 4 weeks (lower panel) stained for ZO-1 (green), CD34 (gray) and DAPI (blue). First line is depicting the three merged channels. Second line is illustrating images of ZO-1 channel alone (gray). On the side, respectively, quantification of ZO-1 fluorescent signal expressed as fold change of control diet. Representative images of a single mouse out of 4-5 mice per group. Scale bar 50 µm; **j**. Relative gene expression levels of junctional proteins after 18 weeks of OSH treatment, expressed as fold change of CD fed group (n=4 mice per group CD, n=10 mice per group HFD and 2% OSH, n=9 mice per group HFD 4% OSH) (*p<0.05; **p<0.01; ***p<0.001; **** <0.0001 one-way ANOVA Dunnett's post-test, line at mean with SEM).

Figure S3

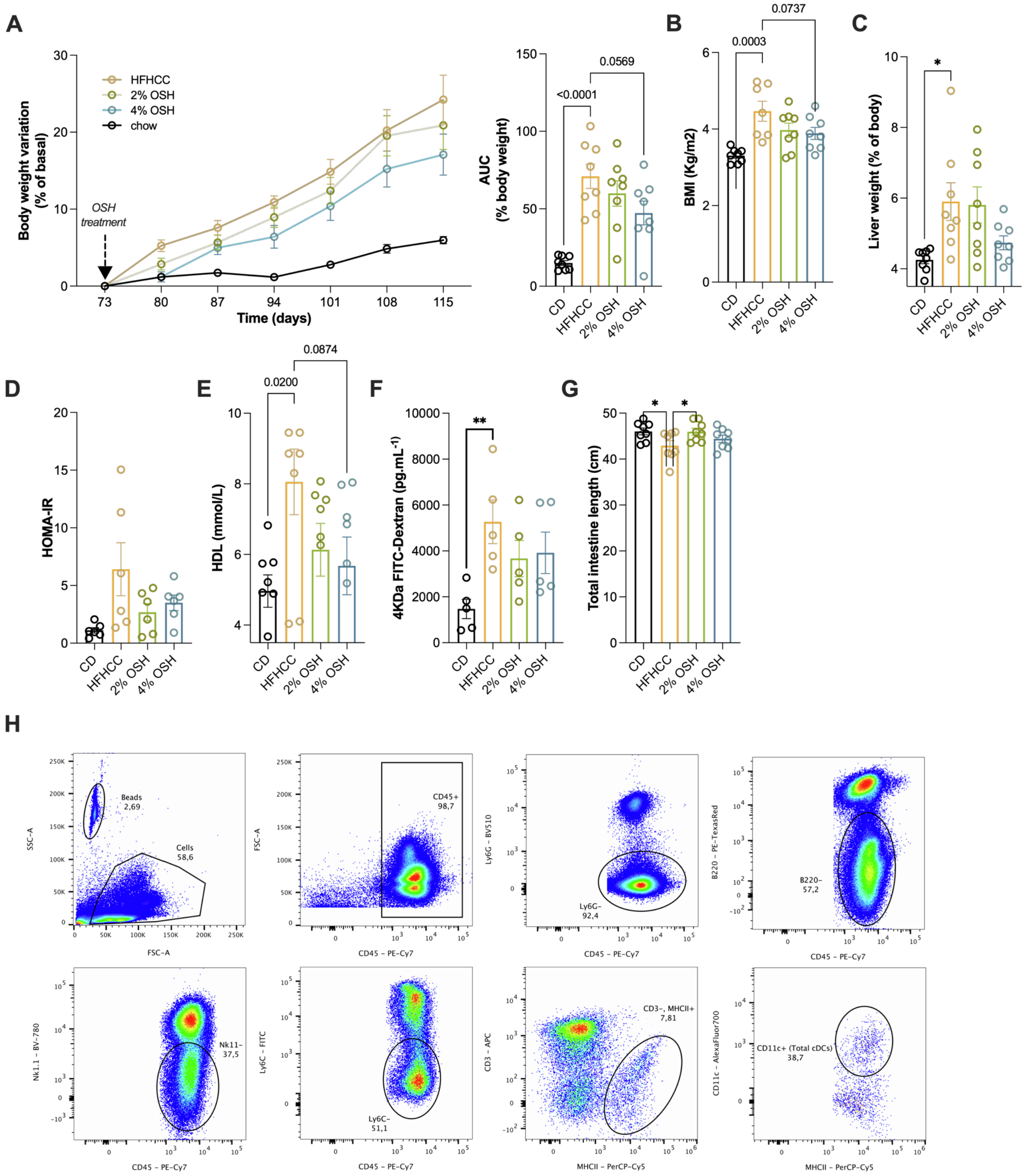

***Supplementary Figure 3*. Effects of therapeutic OSH feeding in NAFLD. Related to Figure 4.**
a. Body weight variation in mice therapeutically fed with OSH after 10 weeks of feeding with HFHCC diet. Body weight is expressed as percentage of body weight at day 73 (before OSH administration) and Area Under the Curve (AUC) of body weight variation curve; **b.** Body Mass Index (BMI) after 6 weeks of therapeutic feeding with OSH, calculated as mouse mass in kg/mouse body length in m^2; **c.** liver weight expressed as percentage of body weight after 6 weeks of therapeutic feeding with OSH; **d.** HOMA-IR (Homeostatic Model Assessment of Insulin Resistance) values after 6 weeks of therapeutic feeding with OSH; **e.** High-Density Lipoprotein (HDL) serum cholesterol levels and after 6 weeks of therapeutic feeding with OSH; **f.**, Serum 4KDa FITC-Dextran, after 6 weeks of therapeutic feeding with OSH supplemented HFHCC diet; **g.** total intestine length after 18 weeks of feeding. **h.** Gating strategy to study liver myeloid cells by flow cytometry, in mice therapeutically fed with OSH supplemented HFHCC for 6 weeks. (*p<0.05; **p<0.01; ***p<0.001; **** <0.0001 one-way ANOVA Dunnett's post-test, line at mean with SEM).

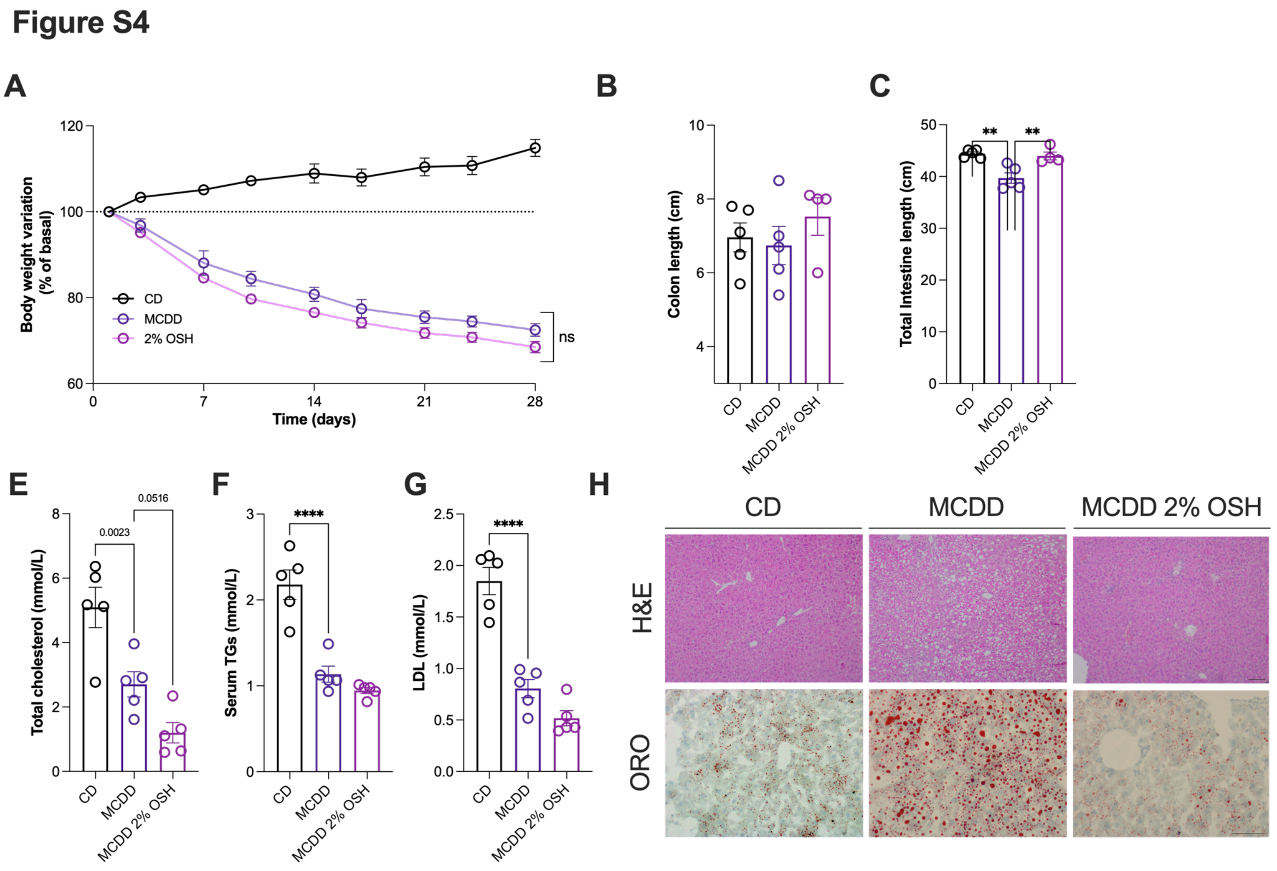

***Supplementary Figure 4*. Effects of preventative OSH administration in a mouse model of NASH. Related to Figure 4.**
C57BL6/J male mice were fed for 4 weeks with MCD diet or MCD diet supplemented with 2 % OSH and control diet (CD). **a.** body weight variation, expressed as percentage of basal body weight; **b.** and **c.** colon length and total intestine length. **e., f.** and **g.** serum total cholesterol, serum total circulating triglycerides and LDL serum cholesterol levels, respectively**,** after 4 weeks of feeding, **h.** liver tissue sections stained for H&E for liver histology (*top line*) and Oil Red O staining for liver triglyceride (*bottom line*), representative images of a single mouse out of 4-5 mice per group, scale bar 100µm. (*p<0.05; **p<0.01; ***p<0.001; **** <0.0001 One-way ANOVA Dunnett's post-hoc test).

Figure S5

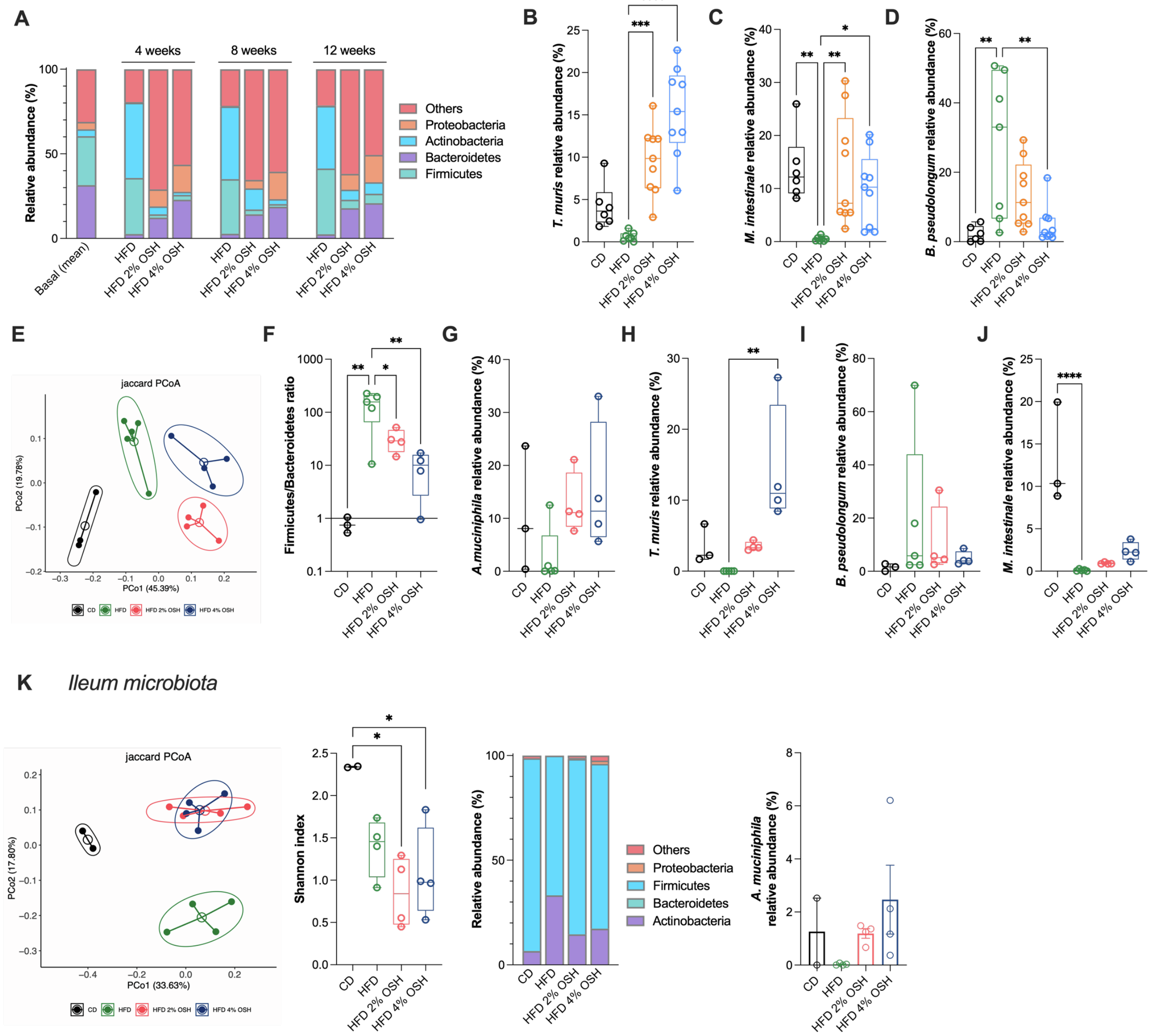

***Supplementary Figure 5*. Gut microbiota modifications induced by preventative and therapeutic OSH administration. Related to Figure 5.**

a. Relative abundance of gut microbiota phyla, evolving in time, throughout 12 weeks preventative OSH administration; **b**.**c.** and **d.** *T.muris, M.intestinale and B.pseudolongum* relative abundance in fecal microbiota of mice fed for 18 week with OSH supplemented HFD (*p<0.05; **p<0.01; ***p<0.001; **** <0.0001 One-way ANOVA Dunnett's post-hoc test); **e.** Principal Coordinate Analysis (PCoA), of fecal microbiota from mice therapeutically fed with OSH supplemented diet for 12 weeks - CD (black), HFD (green) and OSH supplemented HFD (2% OSH pink and 4% OSH blue), based on Jaccard index, single dots represent individual mice (PERMANOVA statistical test, p<0,001); **f.** *Firmicutes/Bacteroidetes* ratio in fecal microbiota of mice therapeutically fed with OSH supplemented diet for 12 weeks; **g.**, **h**. **i.** and **j.** *A. muciniphila*, *T.muris, B.pseudolongum* and *M.intestinale* relative abundance in fecal microbiota of mice therapeutically fed with OSH supplemented diet for 12 weeks (*p<0.05; **p<0.01; ***p<0.001; **** <0.0001 One-way ANOVA Dunnett's post-hoc test); **k.** Analysis of ileum content microbiota of mice therapeutically fed with OSH supplemented diet for 12 weeks, Principal Coordinate Analysis (PCoA), based on Jaccard index, single dots represent pooled samples from individual mice (5 pooled samples for each dot for CD; 3-1-2 and 3 samples for HFD; 3-3-2 and 2 samples for 2% OSH and 3-3-3 and 1 samples for 4% OSH); Shannon's diversity index of ileum microbiota (*p<0.05; One-way ANOVA Dunnett's post hoc test); Relative abundance of detected fecal bacteria phyla expressed as percentage of total; *A.muciniphila* relative abundance in ileum content microbiota of mice therapeutically fed with OSH supplemented diet for 12 weeks.

Figure S6

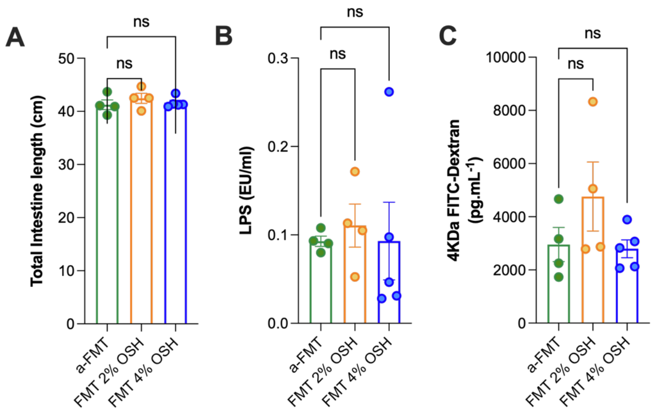

***Supplementary Figure 6*. FMT recipients' metabolic parameters. Related to Figure 7.**
a. Total intestine length of recipient mice receiving FMT every other day for 4 weeks; **b.** Circulating LPS levels of recipient mice receiving FMT every other day for 4 weeks; **c.** Plasma FITC-Dextran (4kDa) levels of recipient mice receiving FMT every other day for 4 weeks (One-way ANOVA Dunnett's post-hoc test).

***Supplementary Table 1*. Animal diet composition. Related to STAR METHODS Experimental Model and Study Participant section.**

	Low Fat control	Standard Chow control	HFD	MCDD	HFHCC
**Code**	TD.86489	4RF21	TD.06415	TD.90262	5TJT
**Supplier**	ENVIGO	MUCEDOLA	ENVIGO	ENVIGO	TESTDIET
**Purified diet**	Yes	No	Yes	Yes	Yes
**Total energy (Kcal/g)**	3.7	3.9	4.6	4.1	4.5
**Protein (%kcal from)**	20.0	18.5	19.0	14.6	16.1
**Carbohydrate (%kcal from)**	67.3	53.5	36.2	63.2	44.2
**Fat (%kcal from)**	12.8	6.0	44.8	22.1	39.7
**Ingredients (g/Kg)**					
**Casein**	207		245		196
**Sucrose**	321		200	455	78
**Starch**	320		85	200	309
**Malto / dextrin**			115		103
**Lard**			195		60
**Oil (soybean or corn)**	30		30	100	18
**Crisco**					60
**Milk Fat**					60
**Cellulose**	50		58	50	25

***Supplementary Table 2*. Primer sequences. Related to STAR METHODS Quantification of DNA and mRNA by Real-Time PCR section.**

**Oligonucleotides**			
**Gene**	**Sequence Fwd**	**Sequence Rev**	**Source**
Slc27a4	ACTGTTCTCCAAGCTAGTGCT	GATGAAGACCCGGATGAAACG	Sigma
Ffar2	CCTCGGTCCAGTGCTGTG	GCCCTGGGACTCGTCAAG	Sigma
Cd36	AGATGACGTGGCAAAGAACAG	CCTTGGCTAGATAACGAACTCTG	Sigma
Fabp6	CTTCCAGGAGACGTGATTGAAA	AACTTGTTGCTCATAATGTTGCC	Sigma
Ppara	AACATCGAGTGTCGAATATGTGG	CCGAATAGTTCGCCGAAAGAA	Sigma
Pparg	GGAAGACCACTCGCATTCCTT	GTAATCAGCAACCATTGGGTCA	Sigma
Cpt1a	CTCCGCCTGAGCCATGAAG	CACCAGTGATGATGCCATTCT	Sigma
Mlxipl	AGATGGAGAACCGACGTATCA	ACTGAGCGTGCTGACAAGTC	Sigma
Fasn	GGAGGTGGTGATAGCCGGTAT	TGGGTAATCCATAGAGCCCAG	Sigma
Cldn1	GAGGGACTGTGGATGTCCTG	ATGCCAATTACCATCAAGGC	Sigma
Cldn2	TTAGCCCTGACCGAGAAAGA	AAAGGACCTCTCTGGTGCTG	Sigma
Cldn3	GAGATGGGAGCTGGGTTGT	GGATCTTGGTGGGTGCATAC	Sigma
Cldn5	AGCTGGTGGCACTCTTTGTT	GCACCGTCGGATCATAGAAC	Sigma
Ocln	CCTCCAATGGCAAAGTGAAT	AGGAATCTCCTGGGCCACT	Sigma
Tjp1	CGCGGAGAGAGACAAGATGT	CCTGTGAAGCGTCAGTGTGT	Sigma
16S rRNA	ACTCCTACGGGAGGCAGCAGT	ATTACCGCGGCTGCTGGC	Sigma
16S rRNA_Amuciniphila	CAGCACGTGAAGGTGGGGAC	CCTTGCGGTTGGCTTCAGAT	Sigma
Muc2	CCTGAAGGCACCACAAAGT	TTGCAGTCAAACTCAAAGT	Sigma
